# Regulation of ribosome hibernation controls *Legionella* survival, infection, antibiotic tolerance, and phenotypic heterogeneity

**DOI:** 10.1128/mbio.03762-25

**Published:** 2026-01-30

**Authors:** Camille Schmid, Selina Natalie Trinkler, Elizabeth Teresa Vittori, Michaela Oborská-Oplová, Vikram Govind Panse, Hubert Hilbi

**Affiliations:** 1 Institute of Medical Microbiology, University of Zürich 27217, Zürich, Switzerland; 2 Faculty of Science, University of Zürich 27217, Zürich, Switzerland; Yale University School of Medicine, New Haven, Connecticut, USA

**Keywords:** *Acanthamoeba*, amoeba, antibiotics, intracellular pathogens, *Legionella*, macrophage, pathogen vacuole, persistence, phenotypic heterogeneity, ribosome hibernation, starvation, stress response, virulence

## Abstract

Bacterial virulence is regulated by the growth phase and ribosome activity, implicating the formation of translationally silent (“hibernating”) ribosomes. *Legionella pneumophila*, the causative agent of Legionnaires’ disease, is a facultative intracellular bacterium that grows in both environmental amoebae and mammalian macrophages. Thus far, ribosome hibernation factors of *L. pneumophila* have not been characterized. Here, we show that *L. pneumophila* encodes homologs of the ribosome hibernating factors LhpF (Lpg1206), RaiA (Lpg0467), RsfS (Lpg1377), and the GTPase HflX (Lpg0010), which define the ribosome populations by mediating 100S ribosome dimerization, 70S inactivation, ribosome assembly inhibition, and ribosome splitting, respectively. Exceptional among γ-proteobacteria, *L. pneumophila* forms 100S ribosome dimers during exponential growth. Functional studies show that the hibernation factors support survival upon starvation, regrowth, efficient host infection, and virulence factor production of *L. pneumophila*. Furthermore, they enhance antibiotic tolerance and shape intracellular heterogeneity of bacterial growth and motility. Our findings identify ribosome hibernation as a central mechanism by which *L. pneumophila* orchestrates survival, persistence, and infection, highlighting its critical role in bacterial physiology and pathogenesis.

**IMPORTANCE:**

Due to nutrient limitation and adverse conditions in the environment, bacteria mostly do not grow exponentially but adopt a resting (“dormant”) state. Bacterial dormancy usually coincides with the formation of translationally silent (“hibernating”) ribosomes; however, the role of ribosome hibernation in intracellular pathogens is poorly understood. The facultative intracellular bacterium *Legionella pneumophila* is virulent in the stationary but not in the exponential growth phase, and therefore, an in-depth characterization of the pathogen’s physiological states and ribosome profiles is crucial for understanding its virulence. Using bioinformatics, bacterial genetics, biochemical, and cell biological approaches, in this study, we reveal that the *L. pneumophila* ribosome hibernation factors LhpF, RaiA, RsfS, and HflX determine distinct ribosome subpopulations and are implicated in starvation survival and regrowth, as well as in host cell infection, intracellular replication, and phenotypic heterogeneity. Collectively, our data highlight the critical importance of ribosome hibernation for the physiology and virulence of *L. pneumophila*.

## INTRODUCTION

*Legionella pneumophila*, the causative agent of Legionnaires’ disease, thrives in both environmental and man-made water systems as planktonic bacteria, in biofilm communities, or within free-living protozoa (1–4). Upon inhalation of contaminated aerosols, the opportunistic pathogen *L. pneumophila* can reach the human lungs and replicate in alveolar macrophages, causing severe and potentially fatal pneumonia (5, 6). *L. pneumophila* utilizes a type IV secretion system (T4SS), known as Icm/Dot (intracellular multiplication/defective organelle trafficking), to translocate over 300 different effector proteins into the host cell, manipulating cellular processes and promoting the formation of the *Legionella*-containing vacuole (LCV), a membrane-bound, ER-associated compartment that serves as the intracellular replication niche (7–14). Given its diverse environmental niches and its ability to persist inside host cells, it is essential for *L. pneumophila* to adopt mechanisms to regulate protein synthesis under stress conditions.

Protein synthesis is one of the most energy-consuming processes in cells, and under stress conditions, bacteria downregulate translation to conserve energy (15, 16). Ribosome “hibernation” is such a strategy, where bacteria form translationally inactive ribosomes as monomers or dimers, thus minimizing energy expenditure while preserving ribosomal integrity (17–20). This mechanism allows bacteria to survive environmental stresses, such as nutrient deprivation, heat, and other adverse conditions, by reducing translation activity and still maintaining the ability to rapidly resume growth. This is essential for bacteria that live in fluctuating environments, such as within biofilms or inside host cells, where nutrients can be irregularly provided or depleted (21, 22).

In most γ-proteobacteria, including *Escherichia coli*, three factors are primarily involved in ribosome hibernation: ribosome modulation factor (RMF), hibernation-promoting factor (HPF, *alias* YhbH), and an HPF paralog called ribosome-associated inhibitor A (RaiA, *alias* YfiA) (23, 24). While RMF and HPF work in concert to dimerize two 70S ribosomes into an inactive 100S dimer, RaiA inactivates ribosomes in the 70S state and antagonizes the formation of 100S dimers (23, 25). Both HPF and RaiA compete for the same binding site at the 30S subunit, where they sterically interfere with the association of the ribosomal decoding center with mRNA, tRNA, and the initiation factors IF1 and IF3 (25–27).

Most other bacteria do not possess RMF or RaiA, but instead, they only utilize a long form of HPF (Long HPF or LhpF) for ribosome hibernation, which also promotes the formation of 100S ribosomes through dimerization (24, 28–30). Compared to short HPF, LhpF possesses a C-terminal extension, which is necessary and sufficient for dimerization (29, 31, 32). Interestingly, while RMF- and HPF-mediated 100S dimers are only present in the stationary growth phase, LhpF-100S ribosomes have been detected throughout all growth phases, indicating that ribosome hibernation can be a constitutive process in some bacterial species (28–30, 33). In addition to forming 100S ribosome dimers, LhpF is also found in the 70S ribosome fraction, potentially stabilizing a second population of hibernating ribosomes as inactivated 70S monomers, similar to RaiA (28, 30, 33). In addition to RMF, HPF, RaiA, and LhpF, another hibernation factor is the ribosomal silencing factor S (RsfS, *alias* RsfA or YbeB). RsfS binds to the 50S subunit, preventing the normal assembly of the 30S and 50S subunits into a functional 70S ribosome (34, 35). This action reduces translation initiation by preventing the formation of new ribosomes.

Following the formation of translationally inactive 70S or 100S ribosomes, it is essential for bacteria to quickly (within minutes) reactivate these ribosomes when conditions improve. The dissociation of these complexes is driven by a conserved GTPase termed high frequency of lysogenization X (HflX), which is responsible for rescuing stalled 70S ribosomes under stress conditions (36). In *S. aureus*, HflX triggers the dissociation of 100S ribosomes in a GTP-dependent manner (37). However, the deletion of HflX does not significantly affect the fraction of dimerized ribosomes, suggesting that other factors also contribute to dissociation.

Ribosome hibernation may play a crucial role in *L. pneumophila* survival and adaptation to stress conditions in different environmental niches. So far, ribosome hibernation factors have not been investigated in *L. pneumophila*. In this study, we identified the *L. pneumophila* ribosome hibernation factors LhpF, RaiA, RsfS, and HflX and show that they impact the bacterial ribosome populations, survival and regrowth after prolonged starvation, as well as virulence and (intracellular) phenotypic heterogeneity.

## RESULTS

### Identification of ribosome hibernation factors in *L. pneumophila*

**Fig 1:**
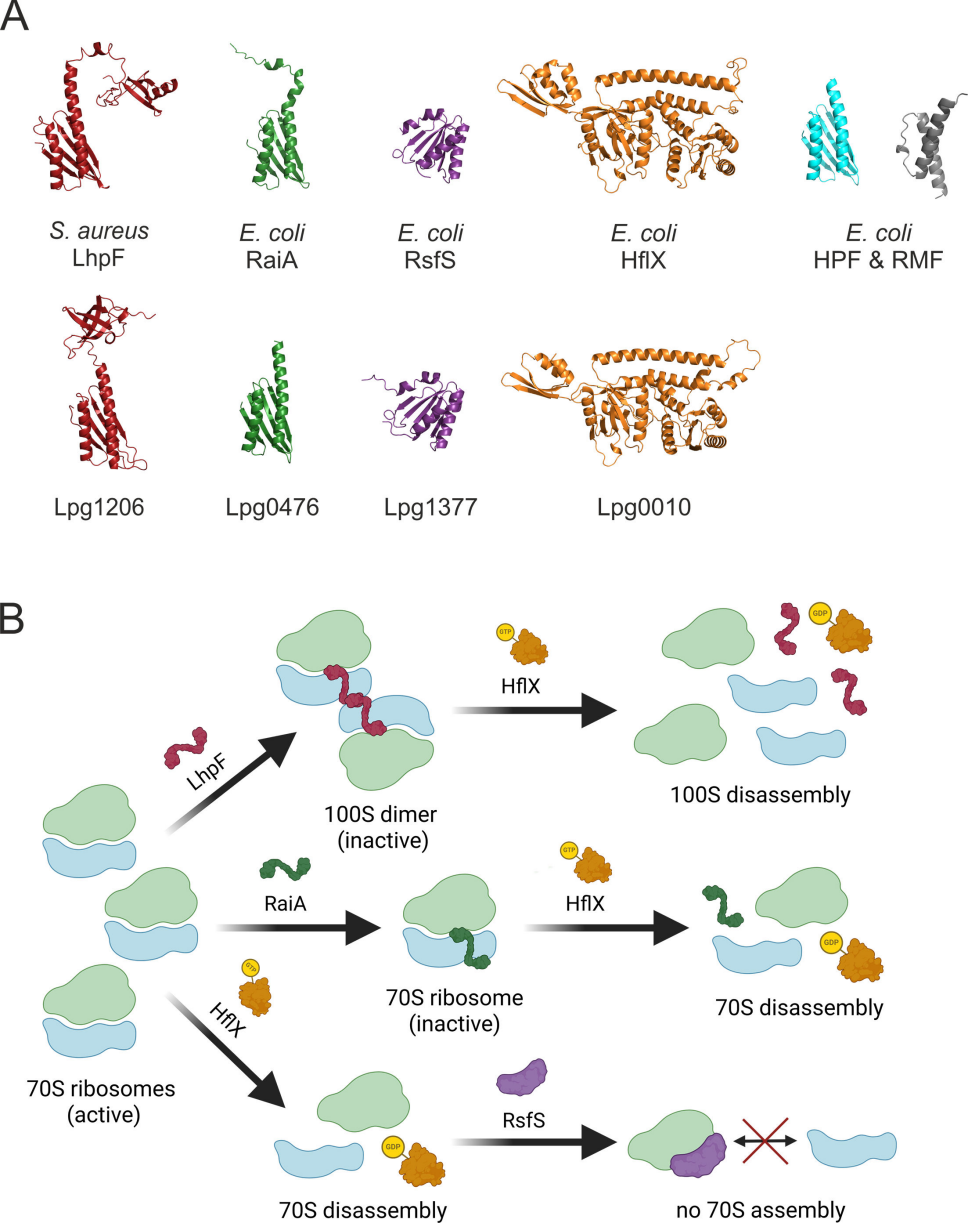
Overview of the ribosome hibernation factors identified in *L. pneumophila* and their proposed modes of action. (**A**) Predicted protein structures of ribosome hibernation factors from *S. aureus*, *E. coli*, and *L. pneumophila*, generated using the AlphaFold Monomer v2.0 pipeline and visualized in PyMOL Molecular Graphics System, v3.0.2. Structural comparisons include the long hibernation promoting factor (LhpF) from *S. aureus* (Q2FIN9) and Lpg1206 from *L. pneumophila* (Q5ZW81; shown in red), the ribosome-associated inhibitor A (RaiA) from *E. coli* (P0AD49) and Lpg0476 (Q5ZY96; green), the ribosome silencing factor S (RsfS) from *E. coli* (P0AAT6) and Lpg1377 (Q5ZVR3; purple), and the GTPase HflX from *E. coli* (P25519) and Lpg0010 (Q5ZZK0; orange). *E. coli* short HPF and ribosomal modulation factor (RMF; P0AFX0 and P0AFW2) are shown in blue and gray, respectively. In *E. coli* and other γ-proteobacteria, 100S ribosome formation is mediated by short HPF and RMF. In contrast, no RMF homolog was found in *L. pneumophila*. (**B**) Schematic representation of proposed mechanisms of ribosome hibernation in *L. pneumophila*. 70S ribosomes can be inactivated through different HPFs. LhpF dimerizes two ribosomes into an inactive 100S dimer while RaiA inactivates ribosomes in the 70S state, both competing for the same binding site on the 30S subunit. RsfS inhibits the subunit association by binding the 50S subunit and blocking the ribosome assembly. The conserved GTPase HflX rescues stalled or inactivated 70S ribosomes and 100S ribosomes by splitting the subunits in a GTP-dependent manner. Created in BioRender. Schmid, C. (2025) https://BioRender.com/x1ym97w.

To identify possible *L. pneumophila* hibernation factors, we searched the proteome of strain Philadelphia-1 using BLASTp. *L. pneumophila* possesses the homologs of both LhpF (Lpg1206) and RaiA (Lpg0467) (Fig. 1A; Fig. S1A and C). The LhpF homolog in *L. pneumophila* shares the N-terminal domain (NTD) of LhpF, short HPF, and RaiA from other species (Fig. S1B and C) but has a distinct C-terminal domain (CTD), differing significantly from LhpF proteins found in organisms such as *Staphylococcus aureus* or *Bacillus subtilis* (Fig. 1A; Fig. S1C). In contrast to the canonical role of LhpF in forming 100S dimers, the distinct C-terminal extension of LhpF in *L. pneumophila* raises questions about its ability to form dimers and whether it might play a different role in ribosome hibernation. Moreover, while most γ-proteobacteria rely on RMF, HPF, and RaiA for ribosome hibernation (18, 30), *L. pneumophila* lacks an RMF homolog and, therefore, presents a unique case. In addition to LhpF and RaiA, *L. pneumophila* encodes a homolog of RsfS (Lpg1377) (Fig. 1A; Fig. S1A and C). Finally, *L. pneumophila* also encodes the conserved GTPase HflX (Lpg0010), responsible for rescuing stalled 70S ribosomes and splitting 100S ribosomes in a GTP-dependent manner (Fig. 1A; Fig. S1A and C). Homologs of most of these putative ribosome hibernation factors were also present in the proteomes of *L. longbeachae*, *L. micdadei*, and *L. anisa* (Fig. S1D).

In summary, based on homologs of known hibernation factors from other bacteria, we identified four distinct ribosome hibernation factors in *L. pneumophila* (Fig. 1A), and we propose three corresponding hibernation mechanisms (Fig. 1B). LhpF promotes the dimerization of two ribosomes into an inactive 100S dimer, RaiA stabilizes ribosomes in an inactive 70S state, and RsfS prevents the assembly of functional 70S ribosomes, effectively silencing ribosomal activity. Finally, the conserved GTPase HflX is thought to dissociate stalled or inactivated 70S and 100S complexes, facilitating their recycling when conditions improve.

### Construction of ribosome hibernation factor mutants in *L. pneumophila*

To assess the role of different ribosome hibernation factors in *L. pneumophila*, we constructed mutant strains by deleting genes encoding the putative ribosome hibernation factors LhpF (*lpg1206*), RaiA (*lpg0476*), RsfS (*lpg1377*), and HflX (*lpg0010*) (Fig. S1A) (for details, see Materials and Methods). We generated single deletion mutant strains (Δ*lhpF*, Δ*raiA*, Δ*rsfS*, and Δ*hflX*), as well as a double mutant (double knock-out, DKO; Δ*lhpF*Δ*raiA*), and all deletions were verified by whole genome sequencing. The DKO strain was constructed due to structural overlap between LhpF and RaiA (Fig. 1A; Fig. S1B), indicating the possibility of functional redundancy and/or competition for the same binding site at the ribosome. Structural similarities and multi-sequence alignments revealed quite a conserved NTD of the *L. pneumophila* LhpF (Lpg1206); however, the CTD is entirely different from other LhpF homologs (Fig. 1A; Fig. S1C).

The growth of the hibernation factor mutant strains was tested in ACES-buffered yeast extract (AYE) broth (rich medium, Fig. S2A) and in minimal defined medium (MDM; Fig. S2B) at different temperatures (AYE: 30°C, 37°C, and 45°C; MDM: 30°C and 37°C). The growth curves showed no significant differences between the mutants and the parental strain in AYE broth, indicating normal growth under rich conditions, even during heat stress. In MDM, only at 30°C, minimal growth defects were observed for the Δ*lhpF* and Δ*rsfS* mutants, as they did not reach the same final cell density as the wild-type strain. We also constructed a *lhpF* booster strain harboring plasmid-encoded *lhpF* under the control of the *raiA* promoter (⁓10× stronger than the native *lhpF* promoter; see below), and a *raiA* overexpression strain harboring plasmid-encoded *raiA* under the control of its native promoter (⁓10× higher expression due to plasmid copy numbers). Growth of the *lhpF* booster (wt pLhpF*) and *raiA* overexpression (wt pRaiA) strains in AYE at 30°C and 37°C was impaired and characterized by a markedly shorter lag phase, especially at 30°C, and moderately reduced final cell densities at both temperatures compared to wild-type bacteria (Fig. S2C).

Taken together, we constructed ribosome hibernation factor mutants in *L. pneumophila*, including single and double deletions, based on their genetic and structural similarities. These mutants were characterized for their growth under different conditions, revealing no significant growth defects in rich medium as well as minor growth defects in minimal medium at 30°C. Overexpression strains of *lhpF* and *raiA* showed moderate growth defects in rich medium at 30°C and 37°C.

### *L. pneumophila* forms 100S dimers in the exponential phase, and its hibernation factors determine the ribosomal population

**Fig 2:**
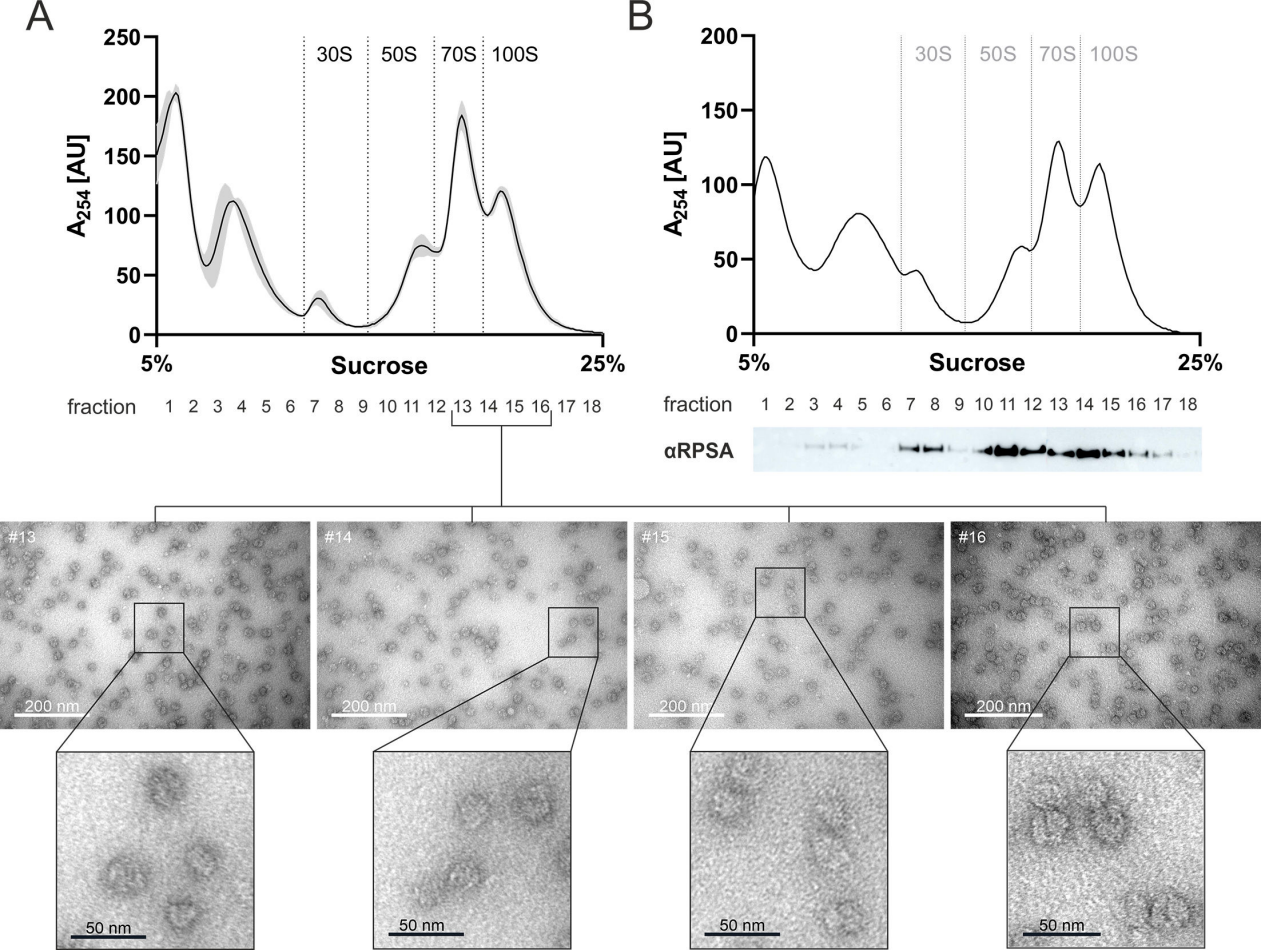
*L. pneumophila* forms 100S ribosome dimers in the exponential phase. (**A, B**) Sucrose gradient profile of ribosomes from virulent *L. pneumophila* JR32 harboring pNT28, harvested as whole cell lysate from an AYE culture in exponential phase grown at 37°C for (**A**) 16 h or (**B**) 18 h. The y-axis corresponds to the absorbance at 254 nm (in arbitrary units, AU) of the ribosome population separated on a 5%–25% sucrose gradient (x-axis). Gradient fractions (eight drops, ~600 µL) were collected and further analyzed via (**A**) negative stain electron microscopy (EM) or (**B**) western blot analysis. (**A**) The ribosomes from fractions 13–16 were visualized by negative stain EM. Scale bar: 200 nm, inset 50 nm. Shown is the riboprofile with the mean and standard deviations (gray shading) of three biological replicates with representative EM pictures of the corresponding fractions (insets: examples of 70S monomers and 100S dimers). (**B**) Gradient fractions were precipitated and separated on a 10% SDS-PAGE gel, and the 30S ribosomal protein RpsA was detected via western blot analysis as a marker for the 30S subunit peak. Shown is one riboprofile with the corresponding western blot representative of three biological replicates.

To assess the contribution of the predicted ribosome hibernation factors to ribosome population dynamics and determine whether *L. pneumophila* forms 100S ribosomes, we performed sucrose gradient profiling of bacterial whole cell lysates (Fig. 2). Visualization by negative stain electron microscopy (EM) of ribosome populations in the sucrose gradient fractions revealed that 100S ribosome dimers were increasingly enriched in later fractions (Fig. 2A). Additionally, we confirmed the peak identities in sucrose gradient fractions by Western blot against RpsA, a ribosomal protein from the small 30S subunit, as a marker for the 30S peak and present in the 70S and 100S ribosome fractions (Fig. 2B).

**Fig 3:**
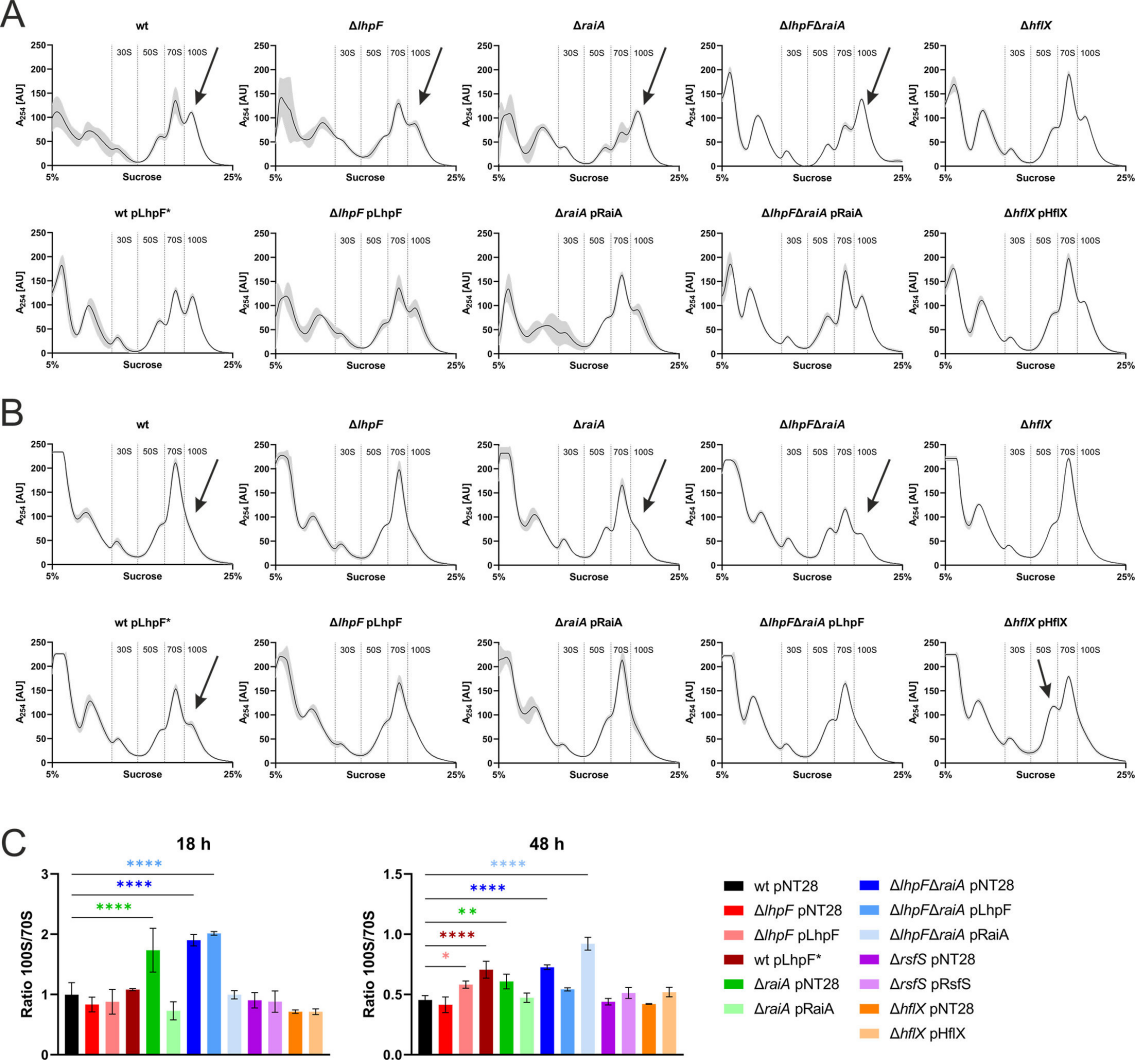
*L. pneumophila* ribosome hibernation factors determine the ribosomal population. (**A, B**) Riboprofiles of *L. pneumophila* JR32, single and double hibernation factor mutants (Δ*lhpF*, Δ*raiA*, Δ*lhpF*Δ*raiA*, and Δ*hflX*), all harboring pNT28, as well as the corresponding complementation strains harboring pLhpF, pRaiA, or pHflX; and the *lhpF* booster strain harboring pLhpF*. Sucrose gradient profiles of ribosomes from *L. pneumophila* whole cell lysates harvested from AYE cultures in (**A**) the exponential phase at 18 h and (**B**) the late stationary phase at 48 h (defined based on OD_600_ measurements, Fig. S3A). The y-axis corresponds to the absorbance at 254 nm (in arbitrary units, AU) of the ribosome population separated on a 5%–25% sucrose gradient (x-axis). Graphs show the means and standard deviations (gray shading) of three biological replicates. Arrows highlight the most significant differences discussed in the main text. (**C**) Quantification of 100S–70S ribosome ratios of all mutant strains analyzed. The area under the curve (AUC) was assessed for each ribosomal sub-population, normalized to the total ribosomal population of the respective profile (Fig. S3D and E), and the ratio of the 100S–70S ribosome populations was calculated. Shown are the means and standard deviations of three biological replicates (*P* < 0.05 *; *P* < 0.01 **; *P* < 0.0001 ****; ANOVA).

Analogously, we performed sucrose gradient profiling of cell lysates of the virulent parental strain JR32, single and double hibernation factor mutants (Δ*lhpF*, Δ*raiA*, Δ*lhpF*Δ*raiA*, Δ*rsfS*, and Δ*hflX*), as well as the complemented strains and the *lhpF* booster strain during exponential and late stationary phases (18 h and 48 h; Fig. 3; Fig. S3). Interestingly, *L. pneumophila* formed 100S dimers preferentially in the exponential phase (Fig. 3A), but not in the stationary phase (Fig. 3B). The identification of the 30S ribosome peak allowed us to detect the 50S, 70S, and 100S peaks, confirming that the 100S dimers are mostly present during the exponential phase. This ribosome distribution is not observed in other bacterial species.

In the Δ*lhpF* strain, the 100S ribosome peak in the exponential phase was smaller but still present, indicating that LhpF contributes to the formation of 100S dimers in *L. pneumophila* but might not be solely responsible. The slight but significant decrease in the percentage of the 100S population was reversed by the presence of the pLhpF complementation plasmid (harboring *lhpF* with its native promoter) (Fig. 3A; Fig. S3D). To further validate the role of LhpF in 100S dimer formation, we constructed a *lhpF* booster strain by fusing the stronger P*_raiA_* promoter (see below) to *lhpF* on a plasmid, which was introduced into the parental strain JR32. This *lhpF* booster strain (wt pLhpF*) showed no increase in 100S ribosomes in the exponential phase, but a clear 100S peak in the late stationary phase, where there was no 100S peak present in the parental strain or in the Δ*lhpF* strain (Fig. 3B and C).

The Δ*raiA* mutant showed a significant increase in the 100S ribosome population during the exponential phase (Fig. 3A and C), which was complemented by the pRaiA plasmid. This finding suggests an at least partially overlapping function of RaiA and LhpF in ribosome hibernation by controlling the formation of 100S dimers. In the late stationary phase, we observed a slight increase in the 100S ribosome population in the Δ*raiA* mutant, which could also be complemented with the pRaiA plasmid (Fig. 3B and C).

The riboprofile of the Δ*lhpF*Δ*raiA* mutant showed that the 100S peak in the exponential phase was still present and comparable to the Δ*raiA* mutant (Fig. 3A and C). This finding reveals that the increase in 100S dimers in the Δ*raiA* strain is not due to LhpF and suggests a yet unidentified factor as a major driver of 100S ribosome formation. This phenotype of the Δ*lhpF*Δ*raiA* mutant was complemented with the pRaiA plasmid, restoring the 100S peak to levels similar to those seen in the parental strain (Fig. 3A and C). In the stationary phase, the Δ*lhpF*Δ*raiA* mutant also displayed slightly more 100S dimers, along with a higher abundance of free 30S subunits compared to the parental strain. This phenotype was complemented with the pLhpF plasmid, which restored the 30S and 100S levels to wild-type values, but increased the levels of free 50S subunits (Fig. 3B and C; Fig. S3E). Intriguingly, the pRaiA plasmid complemented the phenotypes of the Δ*lhpF*Δ*raiA* mutant in the exponential phase but not the stationary phase, while the opposite was observed for pLhpF (Fig. 3; Fig. S3B and C).

In the Δ*rsfS* mutant strain, no significant differences in riboprofiles were observed compared to the parental strain JR32 (Fig. 3C; Fig. S3B through E). The Δ*hflX* mutant also showed no difference in the riboprofiles, neither in the exponential nor in the stationary growth phase (Fig. 3). However, when the Δ*hflX* strain was complemented with the pHflX plasmid, which likely leads to an overexpression of *hflX* due to the plasmid copy number, a significant increase in free 50S ribosome subunits was observed in the stationary phase (Fig. 3B; Fig. S3E). This observation suggests that more 70S ribosomes were split, likely due to HflX activity.

In summary, *L. pneumophila* forms 100S ribosome dimers preferentially in the exponential but not in the stationary growth phase. *L. pneumophila* exhibits a functional redundancy between LhpF and RaiA in controlling 100S formation or 70S inactivation, and, although LhpF plays a role in 100S formation, the similarity of the Δ*raiA* and Δ*lhpF*Δ*raiA* riboprofiles suggests the existence of a yet unidentified factor as a major driver of 100S ribosome formation in the exponential phase. The Δ*rsfS* and Δ*hflX* mutants showed no significant changes in their riboprofiles, but overexpression of *hflX* increased the portion of 50S ribosomes, in agreement with the role of the putative GTPase in ribosome splitting.

### *L. pneumophila* ribosome hibernation factors are implicated in long-term starvation survival and regrowth

**Fig 4:**
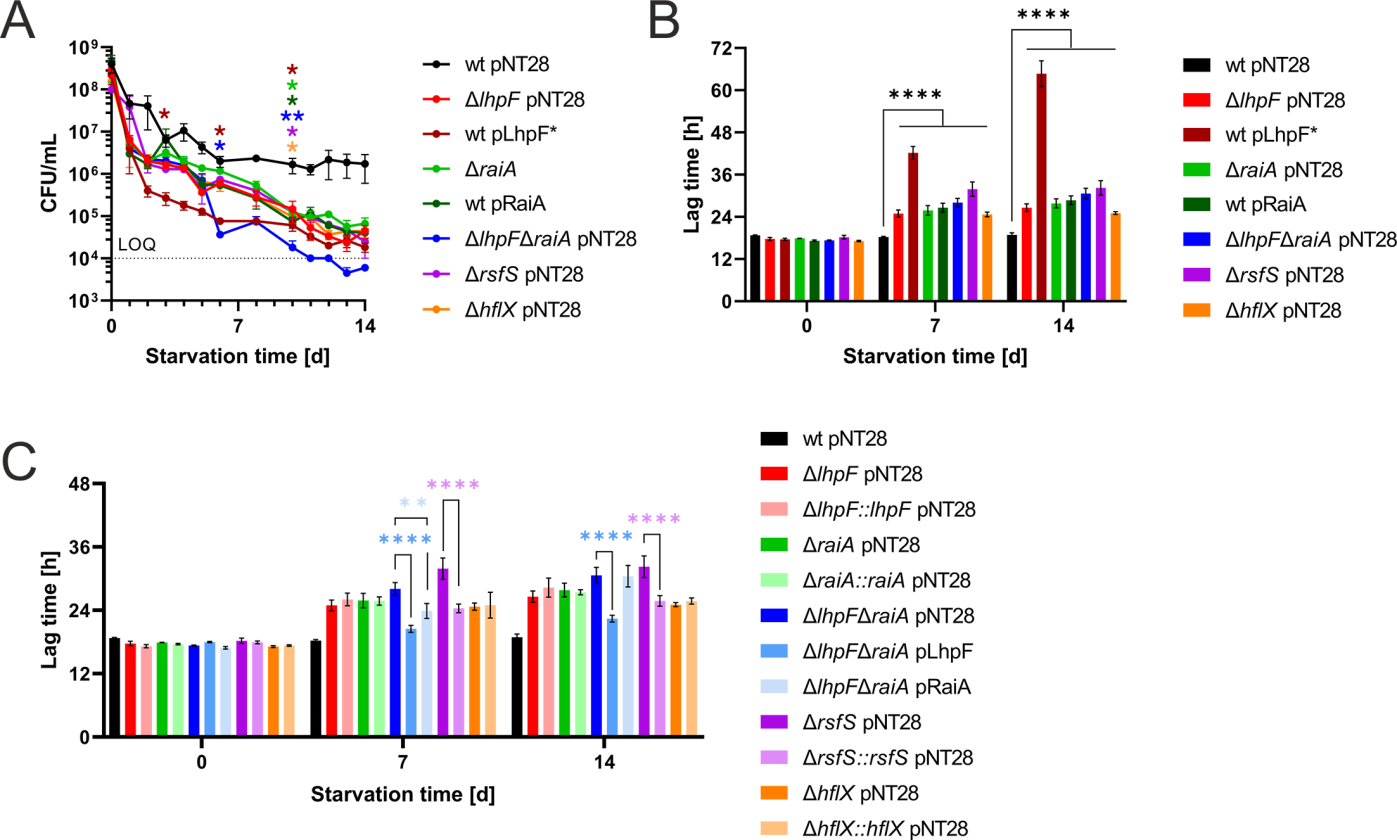
*L. pneumophila* ribosome hibernation factors are implicated in long-term starvation survival and regrowth. *L. pneumophila* JR32, single and double hibernation factor mutants (Δ*lhpF*, Δ*raiA*, Δ*lhpF*Δ*raiA*, Δ*rsfS*, and Δ*hflX*) and the corresponding complemented strains, all harboring either pNT28 or pLhpF or pRaiA, as well as the *lhpF* booster and *raiA* overexpression strains harboring pLhpF* or pRaiA, respectively, were exposed to ACES-buffered ddH_2_O at 37°C for up to 14 days and analyzed for (**A**) culturability and (**B, C**) regrowth in rich medium. In (**A, B**), culturability and regrowth of the wild-type, single and double mutants, and overexpression strains are shown, while in (**C**), regrowth of the wild-type, single mutants and their corresponding reintegration strains and the double mutant and its plasmid-based complementation strains are shown. (**A**) Appropriate dilutions were analyzed for their culturability at different time points by assessing the colony-forming units (CFU). The limit of quantification (LOQ) is indicated by a dashed line. Pseudo values below the LOQ were assigned to *L. pneumophila* strains that were not quantifiable below the LOQ but still detectable. Shown are the means and standard errors of the means of technical triplicates, representative of at least three biological replicates (*P* < 0.05 *; *P* < 0.01 **; two-way ANOVA on Log_10_-transformed data; shown for d3, d6, and d10). (**B, C**) Regrowth after starvation was assessed by inoculating the *L. pneumophila* strains 1:10 into rich medium (AYE) after 0, 7, and 14 days of starvation and growing at 37°C, and OD_600_ was measured for 72 h in a microplate reader. Bar graphs depict the lag time of the strains at the different time points tested. Shown are the means and standard deviations of three biological replicates (*P* < 0.01 **; *P* < 0.0001 ****; two-way ANOVA).

Ribosome hibernation protects ribosomes during stress, and therefore, we assessed the role of hibernation factors in *L. pneumophila* long-term starvation survival and subsequent regrowth. To this end, we used *L. pneumophila* JR32, single and double hibernation factor mutants (Δ*lhpF*, Δ*raiA*, Δ*lhpF*Δ*raiA*, Δ*rsfS*, and Δ*hflX*), as well as complemented strains (chromosomal re-integrations of the corresponding genes; for details, see Materials and Methods) and the booster/overexpression strains (wt pLhpF* and wt pRaiA). The strains were exposed to ACES-buffered H_2_O at 37°C for up to 14 days and analyzed for their culturability and regrowth potential (Fig. 4).

In the long-term starvation assay, all hibernation factor mutant strains displayed worse culturability compared to the parental strain (Fig. 4A). The *lhpF* booster strain showed significantly reduced culturability already in the short term, dropping below the other strains by day 2. The Δ*lhpF*Δ*raiA* mutant exhibited the worst overall culturability, with a marked drop to levels similar to the *lhpF* booster strain around day 7, followed by an even further decline in the second week. Overall, the parental strain JR32 showed a log 2 reduction in colony-forming units (CFU) over the 14-day starvation period, while the Δ*lhpF*, Δ*raiA*, Δ*rsfS*, and Δ*hflX* mutants and the booster/overexpression strains exhibited a log 4 reduction in CFU, and the Δ*lhpF*Δ*raiA* mutant strain displayed nearly a log 5 reduction.

To assess the ability of *L. pneumophila* to regrow after prolonged starvation, the parental and mutant strains were inoculated in rich medium (AYE) after 0, 7, and 14 days of starvation; regrowth was monitored for up to 72 h, and the lag times were analyzed (Fig. 4B). Immediately at the start of starvation, all strains exhibited similar lag times, indicating no differences in their regrowth potential. After 7 days of starvation, all mutants showed a significantly longer lag time than the wild-type strain. Notably, the *lhpF* booster strain displayed the longest lag time and took approximately two times longer to regrow compared to the others. This pattern persisted and was even more pronounced after 14 days of starvation.

To complement the observed phenotypes, chromosomal re-integrations of the deleted genes were constructed in the single mutant strains, and the complemented strains were assessed for their regrowth potential after starvation (Fig. 4C). Using this approach, the ∆*rsfS::rsfS* reintegration strain partially complemented the regrowth defect of the ∆*rsfS* strain, while the other reintegration strains did not complement the phenotypes of the single mutants. In addition, the Δ*lhpF*Δ*raiA* double mutant strain harboring a plasmid carrying either the *lhpF* or *raiA* gene (pLhpF or pRaiA) was assessed (Fig. 4C). Only the Δ*lhpF*Δ*raiA* mutant strain harboring pLhpF complemented the double mutant phenotype, whereas the pRaiA plasmid did not. Taken together, ribosome hibernation factors are implicated in culturability of *L. pneumophila* upon long-term starvation, with the Δ*lhpF*Δ*raiA* mutant showing the most severe survival defects. Although all mutants showed delayed regrowth after starvation, the overexpression of *lhpF* drastically impacted regrowth, indicating that the overexpression of this gene is more detrimental than the deletion of *lhpF* alone or *lhpF* and *raiA* together.

While only the chromosomal reintegration of *rsfS* partially complemented the regrowth defect of the corresponding ∆*rsfS* mutant strain (Fig. 4C), the riboprofiles of the ∆*lhpF*, ∆*raiA*, and ∆*hflX* mutants were complemented by the corresponding plasmid-borne genes (Fig. 3A through C). Given that all mutant strains were verified by whole genome sequencing, these observations are in agreement with a tight dose-, time-, and phenotype-dependent control of the ribosomal hibernation factors in *L. pneumophila*.

### Ribosome hibernation factors promote uptake and intracellular replication of *L. pneumophila*

**Fig 5:**
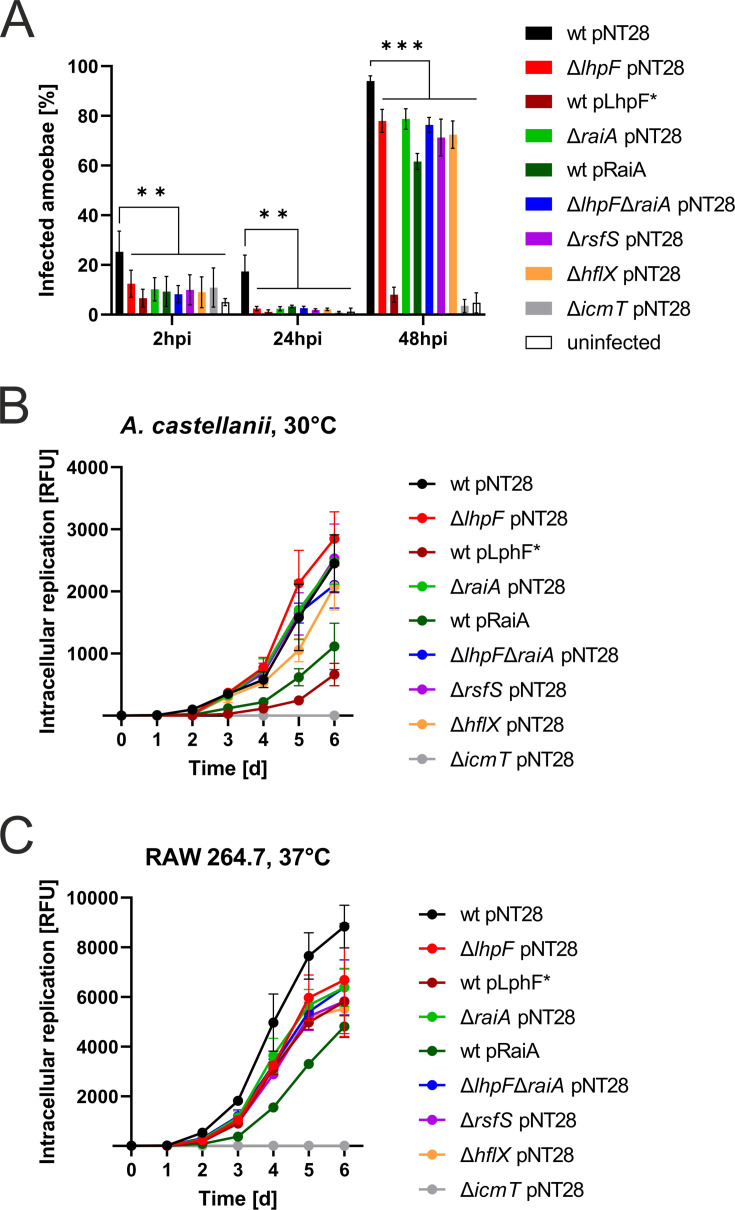
Ribosome hibernation factors promote uptake and intracellular replication of *L. pneumophila*. (**A**) *A. castellanii* amoebae were infected (MOI 1, 2 h, 24 h, 48 h) with GFP-producing *L. pneumophila* JR32, single and double hibernation factor mutants (Δ*lhpF*, Δ*raiA*, Δ*lhpF*Δ*raiA*, Δ*rsfS*, and Δ*hflX*), and the avirulent mutant ∆*icmT,* all harboring pNT28, as well as the *lhpF* booster and *raiA* overexpression strains harboring pLhpF* or pRaiA, respectively. Uninfected amoebae served as controls. Infection rate was assessed by flow cytometry of fixed amoebae. The flow cytometry data are depicted as bar graphs showing the percentage of infected amoebae. Shown are the means and standard deviations of three biological replicates (*P* < 0.01 **; *P* < 0.001 ***; two-way ANOVA). (**B, C**) GFP-producing *L. pneumophila* JR32, single and double hibernation factor mutants (Δ*lhpF*, Δ*raiA*, Δ*lhpF*Δ*raiA*, Δ*rsfS*, and Δ*hflX*), and the avirulent mutant ∆*icmT,* all harboring pNT28, as well as the *lhpF* booster and *raiA* overexpression strains harboring pLhpF* or pRaiA, respectively, were exposed to (**B**) *A. castellanii* amoebae at 30°C (MOI 1, 1-6 days) or (**C**) RAW 264.7 macrophages at 37°C (MOI 1, 1-6 days). Intracellular replication was assessed by relative fluorescence units (RFU). Shown are the means and standard deviations of three biological replicates (bar graphs of specific infection times and statistics shown in Fig. S5A and B).

Given the central role of translational control in coordinating bacterial adaptation during infection, we investigated whether ribosome hibernation factors influence *L. pneumophila* virulence and its intracellular life cycle. To this end, we infected *A. castellanii* amoebae or RAW 264.7 macrophages at 30°C or 37°C with GFP-producing *L. pneumophila* JR32, single or double hibernation factor mutants (Δ*lhpF*, Δ*raiA*, Δ*lhpF*Δ*raiA*, Δ*rsfS*, or Δ*hflX*), or with the booster/overexpression strains (wt pLhpF* or wt pRaiA). As a negative control, the avirulent Δ*icmT* mutant lacking a functional Icm/Dot T4SS was used (Fig. 5). The uptake and infection rates were analyzed at 2, 24, and 48 h post-infection (hpi) by flow cytometry of fixed amoebae (Fig. 5A; Fig. S4). The intracellular replication rates were assessed by infecting host cells with the different *L. pneumophila* strains in replication-limiting medium for up to 6 days and monitoring the increase of GFP signal, translating to intracellular bacterial growth (Fig. 5B and C; Fig. S5).

At 2 hpi, the uptake efficiency of all mutants was lower than that of the parental strain (Fig. 5A). Specifically, ca. 25% of the amoebae were infected with the parental strain, while only ca. 10% of the amoebae were infected with a mutant, indicating roughly a 2.5-fold reduction in uptake. All ribosome hibernation mutants (and the overexpression/booster strains) displayed uptake levels comparable to the Δ*icmT* strain, which is impaired for uptake due to the loss of a functional Icm/Dot T4SS (38).

At 24 hpi, we observed a marked reduction in the percentage of infected amoebae compared to 2 hpi (Fig. 5A; Fig. S4), reflecting the bacteria’s ability to survive and replicate inside the host cell. For the virulent parental strain, approximately 17% of amoebae were infected at 24 hpi, meaning nearly 70% of the bacteria that were taken up successfully established an LCV and started replicating. In contrast, the ribosome hibernation mutants showed only around 2% infected amoebae at 24 hpi, corresponding to only ~20% of the bacteria successfully forming an LCV after uptake. This reduction in successful LCV formation underscores the importance of the ribosome hibernation factors for establishing infection in *A. castellanii*.

At 48 hpi, the parental strain reached 95% infected amoebae, while the mutant strains exhibited only 70%–80% infection, reflecting a second round of infection and intracellular replication (Fig. 5A; Fig. S4). The *lhpF* booster strain showed the most severe infection phenotype, with only 8% infected amoebae, while the *raiA* overexpression strain exhibited ⁓60% infected amoebae, indicating a significant reduction in virulence in these strains. The Δ*icmT* strain did not establish an infection, remaining at 3% infected amoebae comparable to the uninfected control. Taken together, these results highlight the importance of ribosome hibernation factors for efficient uptake and intracellular replication of *L. pneumophila* and indicate that the overexpression of *lhpF* or *raiA* is detrimental to *L. pneumophila*’s ability to infect and replicate effectively within *A. castellanii*.

To follow the intracellular replication over a longer time course and in different host cells, we infected *A. castellanii* or RAW 264.7 macrophages and monitored intracellular replication for up to 6 days (Fig. 5B and C; Fig. S5). For *A. castellanii* infected at 30°C (Fig. 5B; Fig. S5A), the early replication dynamics observed by flow cytometry were replicated. On day 2 of the infection, all mutants or booster/overexpression strains showed an impaired intracellular replication compared to the wild-type strain (Fig. S5A). On day 3, the mutant strains caught up to the parental strain and showed the same replication dynamics for the remainder of the experiment. However, the strains overexpressing either *lhpF* or *raiA* never caught up and, instead, lagged behind the other strains through day 6, with the *lhpF* booster strain showing the most severe intracellular replication defect. For RAW 264.7 macrophages infected at 37°C (Fig. 5C; Fig. S5B), we observed quite different intracellular replication dynamics. From day 2 onward, all mutant strains showed a clear reduction in intracellular replication, and none of the strains ever caught up to wild-type levels. Most strikingly, however, the *lhpF* booster strain no longer showed the most severe virulence defect but replicated at the same rate as the mutant strains. Only the *raiA* overexpression strain still showed a more pronounced intracellular replication phenotype.

Based on these observations, we sought to assess whether the infection phenotype of the *lhpF* booster strain is host cell-dependent or temperature-dependent. To this end, we repeated the infection of *A. castellanii* at 37°C, where the amoebae are slightly stressed by elevated temperature, and the *L. pneumophila* strains replicate faster (Fig. S5C and D). Under these conditions, we no longer observed any infection phenotypes for the ribosome hibernation mutant strains. Only the booster/overexpression strains showed a decreased intracellular replication on day 2, with the *raiA* overexpression strain continuously lagging behind all other strains through day 6. However, from day 3 onward, the *lhpF* booster strain caught up to the wild-type strain and no longer displayed any virulence defects, suggesting that the observed differences between the host cell types were indeed temperature-dependent, with the *lhpF* booster strain displaying a temperature-sensitive virulence phenotype.

In summary, ribosome hibernation factors are crucial for the uptake and intracellular replication of *L. pneumophila*. The hibernation factor mutant strains showed reduced uptake and replication rates in *A. castellanii* and RAW 264.7 macrophages, while the overexpression of *lhpF* and *raiA* caused severe virulence defects, with *lhpF* showing a temperature-dependent infection phenotype.

### Transcriptional regulation of *L. pneumophila* ribosome hibernation factors

**Fig 6:**
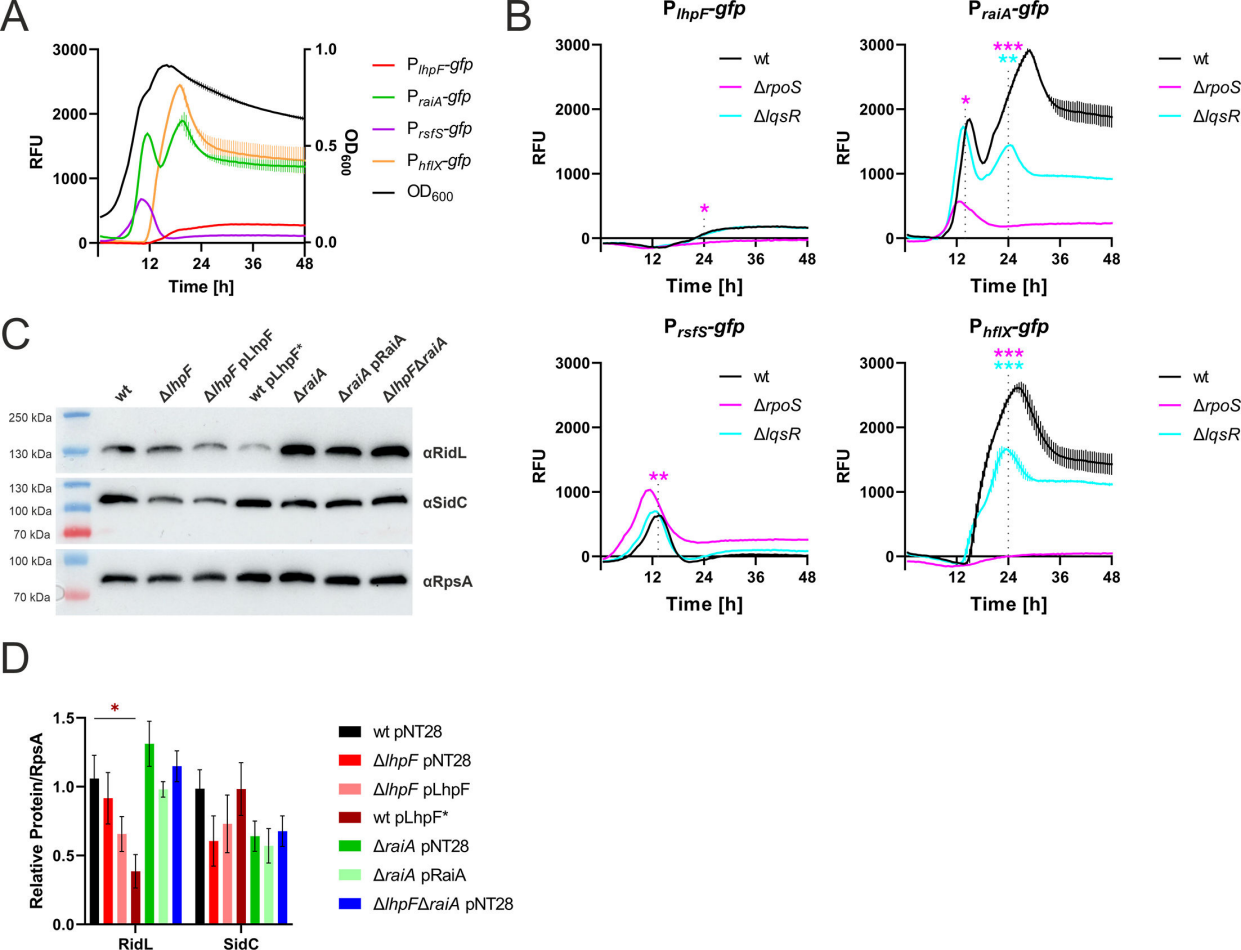
Transcriptional regulation of ribosome hibernation factors and their role in *L. pneumophila* effector protein production. (**A**) *L. pneumophila* JR32 or (**B**) JR32, ∆*rpoS* and ∆*lqsR* harboring P*_lhpF_-gfp* (pCS012), P*_raiA_-gfp* (pCS017), P*_rsfS_-gfp* (pCS018), or P*_hflX_-gfp* (pCS019) were grown in AYE medium at 37°C. OD_600_ and GFP fluorescence were measured over time using a microplate reader. Promoter activity is inferred *via gfp* expression levels, denoted as relative fluorescence units (RFU, left y-axis), and one representative OD_600_ curve (right y-axis). Shown are the means and standard deviations of technical triplicates, representative of at least three biological replicates (*P* < 0.05 *; *P* < 0.01 **; *P* < 0.001 ***; two-way ANOVA; shown for 14 h and/or 24 h). Some error bars may not be visible because they are too small and hidden by the connecting line. (**C, D**) Lysates of late stationary phase of *L. pneumophila* JR32, single and double hibernation factor mutants (Δ*lhpF*, Δ*raiA*, and Δ*lhpF*Δ*raiA*) harboring pNT28, as well as the corresponding complementation strains harboring the plasmids pLhpF or pRaiA, respectively, and the *lhpF* booster strain harboring pLhpF*, were separated by SDS-PAGE and analyzed by anti-RidL, anti-SidC, and anti-RpsA western blots. (**C**) Western blots of one representative sample and (**D**) quantification of (**C**). Signal intensities of RidL, SidC, and RpsA were calculated by Image J. Bars show relative RidL and SidC intensities normalized to RpsA. Shown are the means and standard errors of the means of three biological replicates. (*P* < 0.05 *; two-way ANOVA).

Next, we sought to assess how *L. pneumophila* fine-tunes ribosome hibernation by transcriptional regulation. To this end, we examined the expression of *L. pneumophila* ribosome hibernation factors in the parental strain JR32 harboring reporter constructs, where the promoters of the ribosome hibernation factors were fused to *gfp* (Fig. 6A). Under optimal growth conditions (AYE medium, 37°C), the *lhpF* promoter displayed weak activity exclusively during stationary growth phase, whereas the *raiA* promoter exhibited approximately 10-fold stronger activity, with pronounced peaks in both the exponential and the stationary phase. The *rsfS* promoter showed intermediate-level activity, restricted exclusively to the exponential phase, while *hflX* promoter activity occurred solely during the stationary phase, reaching levels similar to the P*_raiA_-gfp* expression.

To assess the impact of temperature and nutrient availability on promoter activity, we compared GFP fluorescence of the reporter strains at different temperatures (30°C, 37°C, and 45°C) in rich AYE medium (Fig. S6A), as well as at 30°C and 37°C in minimal defined medium (MDM) (Fig. S6B). While the *lhpF* promoter already showed very low activity even under optimal conditions, at elevated temperature (45°C), it was further reduced (~2-fold lower), and the activity was completely abolished at the lower temperature (30°C). Under nutrient-limiting conditions (MDM), *lhpF* promoter activity was comparable to optimal conditions at 30°C but barely detectable at 37°C. The *raiA* promoter, highly active in exponential and stationary phases at optimal growth, displayed approximately 2-fold lower activity at 30°C and a pronounced loss of the stationary phase peak at 45°C (~3-fold reduction). In nutrient-limited MDM medium, *raiA* promoter activity was characterized by the loss of the stationary-phase peak and an overall ~2-fold reduction at 30°C and a more pronounced reduction (~5-fold) at 37°C compared to optimal conditions. The *rsfS* promoter showed stable exponential phase activity, which was reduced (~2-fold) at a lower temperature (30°C), but increased by approximately 2-fold at an elevated temperature (45°C). Interestingly, *rsfS* promoter activity was stable in MDM at both 30°C and 37°C, indicating that *rsfS* expression is induced at higher temperatures and is less sensitive to nutrient limitation. The *hflX* promoter, active solely in the stationary phase, showed significantly reduced activity (~5-fold reduction) at a lower temperature (30°C) compared to optimal growth conditions, and its expression was drastically decreased (~20-fold reduction) under heat stress (45°C). Under nutrient-limiting conditions (MDM), the *hflX* promoter displayed reduced stationary-phase activity (~5-fold reduction) at 30°C, while at 37°C, its activity was nearly undetectable, with a very late stationary phase expression (~20-fold reduction compared to optimal conditions).

To investigate the roles of the *L. pneumophila* stationary phase sigma factor RpoS and the Lqs quorum-sensing system response regulator LqsR, both key regulators of the growth phase switch and the expression of transmission/virulence traits, we measured promoter activity in Δ*rpoS* and Δ*lqsR* backgrounds at 37°C in AYE medium (Fig. 6B). Compared to the expression in the parental strain, the *lhpF* promoter showed no detectable activity in the Δ*rpoS* mutant, indicating a complete dependence on RpoS, whereas the activity remained unchanged in the Δ*lqsR* mutant. The *raiA* promoter showed a ca. 3-fold reduction of activity in the exponential phase in Δ*rpoS,* along with a complete loss of activity in the stationary phase, and a ca. 2-fold reduction in activity in the stationary phase in Δ*lqsR*. While the *rsfS* promoter activity remained unaffected in Δ*lqsR*, it slightly increased (~1.5-fold induction) in Δ*rpoS* compared to wild-type. Finally, *hflX* promoter activity exhibited a moderate (~1.5-fold) reduction in Δ*lqsR* and was completely abolished in the Δ*rpoS* mutant, highlighting the critical role of RpoS in regulating its expression. In summary, these observations indicate that the *L. pneumophila* ribosome hibernation factors LhpF, RaiA, RsfS, and HflX show distinct and complex expression profiles regulated by temperature, nutrient availability, and key regulatory proteins such as RpoS and, to a somewhat lesser extent, LqsR.

### Role of *L. pneumophila* ribosome hibernation factors for effector protein production

Given the infection defects observed for ribosome hibernation mutants (Fig. 5) and the strong regulation of all hibernation factors by RpoS and LqsR (Fig. 6B), two key regulators of virulence traits, we next investigated whether ribosome hibernation factors influence the production of virulence factors (effector proteins). To this end, we assessed the *L. pneumophila* ribosome hibernation factor mutant and overexpression strains, which showed riboprofiles different from the parental strain (Fig. 3; Fig. S3), and performed western blots against the well-characterized effectors RidL (39) and SidC (40) in the lysates of late stationary phase bacterial cultures (Fig. 6C and D). RidL, which is produced in both the exponential and stationary growth phases (41), was slightly less abundant in the Δ*lhpF* mutant compared to wild-type *L. pneumophila* and further reduced in the *lhpF* booster strain, whereas it was present in slightly higher amounts in the Δ*raiA* mutant. SidC, which is only expressed in the stationary growth phase (41), was produced at similar levels in the different *L. pneumophila* strains, although the trends indicated a slightly reduced production in the Δ*lhpF*, Δ*raiA*, and the Δ*lhpF*Δ*raiA* mutants. Taken together, the *L. pneumophila* ribosome hibernation factors play a role in regulating the production of virulence factors (effector proteins), such as RidL and SidC.

### Ribosome hibernation factors modulate antibiotic tolerance and phenotypic heterogeneity of growth and motility

**Fig 7:**
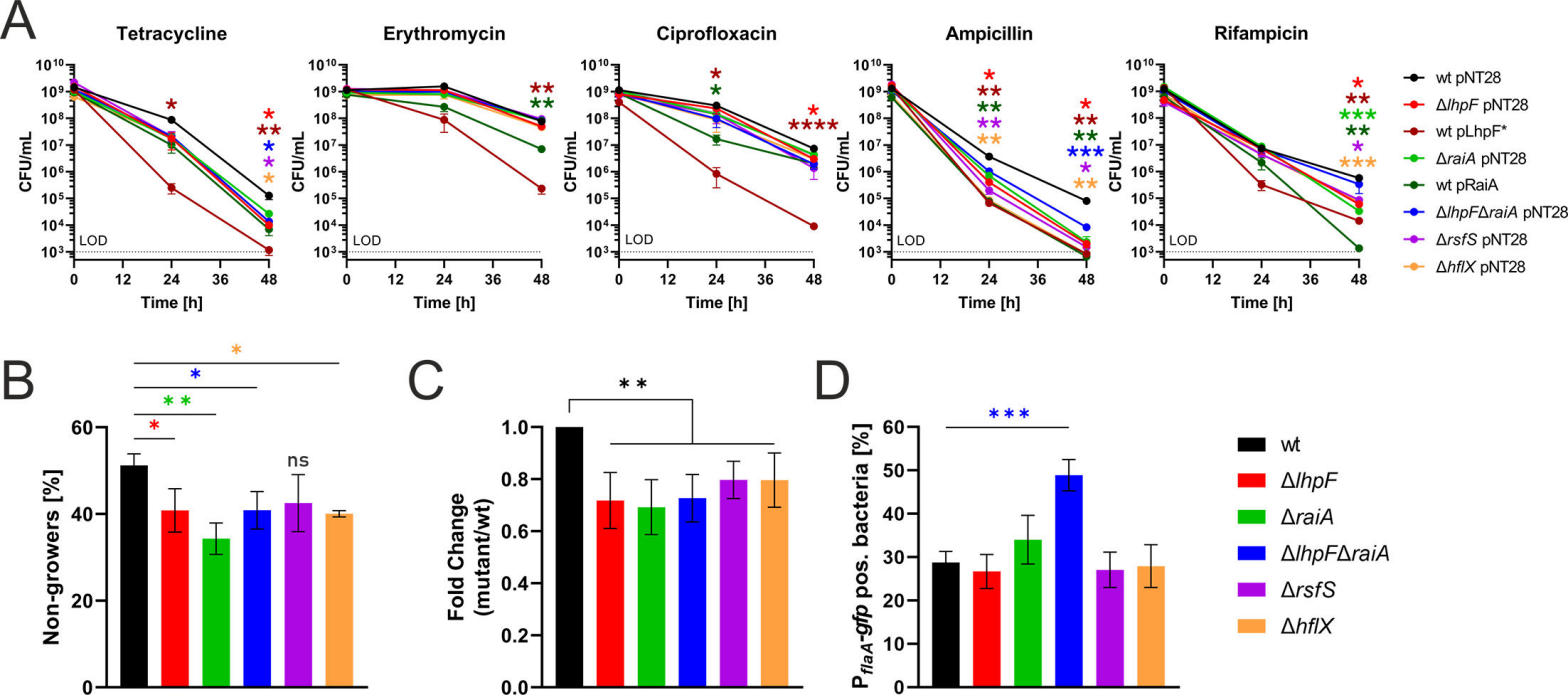
Ribosome hibernation factors enhance antibiotic tolerance and modulate phenotypic heterogeneity of growth and motility. (**A**) *L. pneumophila* JR32, single and double hibernation factor mutants (Δ*lhpF*, Δ*raiA*, Δ*lhpF*Δ*raiA*, Δ*rsfS*, and Δ*hflX*) harboring pNT28, as well as the *lhpF* booster and *raiA* overexpression strains harboring pLhpF* or pRaiA, respectively, were assessed for antibiotic tolerance by exposing the bacteria to 100 µg/mL tetracycline, 100 µg/mL erythromycin, 3.2 µg/mL ciprofloxacin, 100 µg/mL ampicillin, or 1 µg/mL rifampicin for up to 48 h. Survival was analyzed by assessing the CFU at 0 h, 24 h, and 48 h, and the limit of detection (LOD) is indicated by a dashed line. Shown are the means and standard errors of the means of technical triplicates, representative of three biological replicates (*P* < 0.05 *; *P* < 0.01 **; *P* < 0.001 ***; *P* < 0.0001 ****; two-way ANOVA on Log_10_-transformed data). (**B**) *A. castellanii* amoebae were infected (MOI 1, 20 h) with Timer-producing *L. pneumophila* JR32, single and double hibernation factor mutants (Δ*lhpF*, Δ*raiA*, Δ*lhpF*Δ*raiA*, Δ*rsfS*, and Δ*hflX*) harboring pNP107. The phenotypic heterogeneity was assessed by flow cytometry in lysates, and data are shown as bar graphs depicting the percentage of non-growing bacteria. Shown are the means and standard deviations of technical triplicates, representative of 4–6 biological replicates (*P* < 0.05 *; *P* < 0.01 **; ANOVA). (**C**) Fold change of (**B**) normalized to the respective values of strain JR32. Shown are the means and standard deviations of 4–6 biological replicates (*P* < 0.01 **; ANOVA). (**D**) *A. castellanii* amoebae were infected (MOI 1, 48 h) with P*_flaA_-gfp*-expressing *L. pneumophila* JR32, single and double hibernation factor mutants (Δ*lhpF*, Δ*raiA*, Δ*lhpF*Δ*raiA*, Δ*rsfS*, and Δ*hflX*) harboring the dual reporter pSN7. The phenotypic heterogeneity was assessed by flow cytometry in lysates and data is shown as bar graphs depicting the percentage of P*_flaA_-gfp*-positive bacteria. Shown are the means and standard deviations of three biological replicates (*P* < 0.001 ***; ANOVA).

Many antibiotics target the bacterial ribosome, and ribosome hibernation has been implicated in increased tolerance not only to ribosome-targeting drugs but also to other antibiotic classes (42, 43). We therefore tested the antibiotic sensitivity of planktonic *L. pneumophila* hibernation mutants toward multiple antibiotic classes. To this end, *L. pneumophila* JR32, single and double hibernation factor mutants (Δ*lhpF*, Δ*raiA*, Δ*lhpF*Δ*raiA*, Δ*rsfS*, and Δ*hflX*), and the booster/overexpression strains (JR32 pLhpF* and JR32 pRaiA) were exposed to ca. 100-fold minimum inhibitory concentration (MIC) of the antibiotics tetracycline, erythromycin, ciprofloxacin, ampicillin, or rifampicin, and the decrease in bacterial CFU was followed over 48 h (Fig. 7A). Notably, the *lhpF* booster strain (JR32 pLhpF*) was drastically more sensitive to all the antibiotics tested. In addition, the *raiA* overexpression strain showed a significantly decreased tolerance toward erythromycin, ciprofloxacin, and rifampicin. All mutants were less tolerant than the parental strain toward tetracycline and ampicillin, with the *lhpF* booster strain again being the most sensitive (Fig. 7A).

Antibiotic tolerance can be due to the occurrence of “persisters,” a population of non-growing, metabolically active bacteria, which is phenotypically less susceptible to antibiotics (44). Accordingly, we sought to assess whether the antibiotic tolerance correlated with the occurrence of intracellular non-growing *L. pneumophila*. To this end, we utilized as a growth rate proxy the Timer reporter, a fluorescent reporter protein that shifts its fluorescence from green to red as it matures (45, 46), allowing for the discrimination between growing and non-growing bacteria. To assess the intracellular populations of growing and non-growing *L. pneumophila*, we infected *A. castellanii* with *L. pneumophila* JR32 or single or double hibernation factor mutants (Δ*lhpF*, Δ*raiA*, Δ*lhpF*Δ*raiA*, Δ*rsfS*, and Δ*hflX*) harboring the Timer system (Fig. 7B and C). Lysates of the infected amoebae were analyzed for green/red Timer fluorescence ratios and gated into growing *versus* non-growing sub-populations to quantify non-growing bacteria (Fig. S7A). Under these conditions, we observed a reduction in the number of non-growers in the ribosome hibernation mutants compared to the parental strain (Fig. 7B). The quantification of the phenotype revealed a significant 20%–30% reduction of non-growing mutants compared to the wild-type strain, as indicated by fold change (mutant/wild-type) values (Fig. 7C). Accordingly, the reduction of non-growing bacteria in the ribosome hibernation mutant strains correlates to a reduction of antibiotic tolerance in these strains.

Another trait shaped by phenotypic heterogeneity is the emergence of a transmissive, flagellated subpopulation of intracellular *L. pneumophila*, promoting LCV egress and host cell exit (47). Using the P*_tac_-mCherry*-P*_flaA_-gfp* dual reporter system, we assessed the heterogeneous expression of the stationary phase promoter P*_flaA_* during the late stages of infection, which determines the transmissive sub-population promoting LCV/host cell exit. To assess the heterogeneous expression of P*_flaA_*, we infected *A. castellanii* with *L. pneumophila* JR32, single or double hibernation factor mutants (Δ*lhpF*, Δ*raiA*, Δ*lhpF*Δ*raiA*, Δ*rsfS*, and Δ*hflX*) harboring the P*_flaA_-gfp* dual reporter (Fig. 7D). Lysates of the infected amoeba cells were analyzed for GFP signal produced by P*_flaA_-gfp* and gated for GFP-positive *versus* GFP-negative bacteria (Fig. S7B). Under the conditions used, we observed a significantly higher percentage of GFP-positive bacteria in the Δ*lhpF*Δ*raiA* DKO mutant (ca. 50%) compared to the wild-type strain and other ribosome hibernation mutants (ca. 30%) (Fig. 7D). Accordingly, LhpF, along with RaiA, appears to restrict the appearance of flagellated, transmissive *L. pneumophila* at late stages of infection. In summary, ribosome hibernation factors appear to regulate intracellular phenotypic heterogeneity in *L. pneumophila*, promoting the formation of intracellular non-growing bacteria and suppressing the emergence of motile bacteria. The ribosome hibernation factors are therefore key for bacterial survival under antibiotic or host-associated stress, impacting persistence, infection, and spread of *L. pneumophila*.

## DISCUSSION

In this study, we identified the *L. pneumophila* ribosome hibernation factors LhpF, RaiA, RsfS, and HflX (Fig. 1; Fig. S1), constructed defined deletion mutant strains, and characterized their role in forming distinct ribosome subpopulations (Fig. 3; Fig. S3), starvation survival and regrowth (Fig. 4), host cell infection and intracellular replication (Fig. 5; Fig. S5), effector protein production (Fig. 6), and antibiotic tolerance and phenotypic heterogeneity (Fig. 7). Collectively, these findings highlight the critical importance of ribosome hibernation for *L. pneumophila* survival, persistence, and virulence.

The pattern and production of ribosome hibernation factors in *L. pneumophila* contrast with other γ-proteobacteria like *E. coli*, which, through the combined action of RMF and short HPF, form 100S ribosome dimers during the stationary phase (23, 24). *L. pneumophila* lacks an RMF homolog and distinctively produces LhpF as well as RaiA (Fig. 1; Fig. S1). Depending on LhpF and RaiA, *L. pneumophila* forms 100S ribosome dimers preferentially in the exponential phase (Fig. 2 and 3). Perhaps these post-translationally inactivated ribosomes allow a rapid translational response at a later (stationary) growth phase, which represents the virulent phase of *L. pneumophila* (48, 49), where many effector proteins are produced (41). In previous proteomics and transcriptomics studies, LhpF was identified in stationary phase *L. pneumophila* (41), and *lhpF* gene expression was found to be significantly upregulated upon exposure of *L. pneumophila* to water (50), suggesting a role of this ribosome hibernation factor in response to nutrient limitation and for stress protection.

Depending on LhpF, *S. aureus* and *Listeria monocytogenes* form 100S ribosomes already during exponential growth, peaking at the transition to stationary phase (28–30, 33). In *S. aureus* and *L. monocytogenes*, LhpF also inactivates ribosomes as 70S monomers (28, 33). As the stationary phase progresses, the proportion of 100S dimers decreases in these pathogens, coinciding with a rise in LhpF-bound, inactive 70S monomers. It has been hypothesized that LhpF-70S complexes might be easier to convert into active 70S ribosomes than the more stable 100S complexes, potentially reflecting dynamic translational needs under changing conditions (28, 33). It is currently unknown whether, in addition to promoting the formation of 100S ribosomes (Fig. 3), *L. pneumophila* LhpF also inactivates 70S ribosomes.

In contrast to other γ-proteobacteria, the *L. pneumophila* hibernation factors were strongly regulated by the alternative (stationary phase) sigma factor RpoS, and more moderately by the quorum-sensing response regulator LqsR (Fig. 6B), the production of which in turn is positively regulated by RpoS (51). Specifically, the activity of the *L. pneumophila lhpF* promoter was strictly dependent on RpoS but independent of LqsR, whereas the *raiA* promoter showed reduced stationary-phase expression in both Δ*rpoS* and Δ*lqsR* backgrounds. While the *rsfS* promoter was induced in the Δ*rpoS* background but independent of LqsR, the promoter of *hflX* was reduced in Δ*lqsR* backgrounds and dependent on RpoS. Together, these results reveal an intricately interwoven regulatory network comprising RpoS, LqsR, and ribosome hibernation factors. In contrast, in *E. coli*, the expression of *rmf*, *hpf,* and *raiA* is independent of RpoS but tightly regulated by (p)ppGpp and cAMP (18, 52–54).

While the *L. pneumophila* Δ*lhpF* strain showed a smaller 100S ribosome peak in the exponential phase, the Δ*raiA* mutant showed a significant increase, and the *lhpF* booster strain (wt pLhpF*) showed a 100S peak in the late stationary phase (Fig. 3; Fig. S3). These results indicate that LhpF and RaiA are implicated in 100S ribosome formation and might compete for the same binding site. Analogously, studies with *E. coli* have shown antagonistic interactions between HPF and RaiA, where one factor promotes, and the other prevents ribosome dimerization (25). Intriguingly, the Δ*lhpF*Δ*raiA* double mutant maintained high levels of 100S ribosome dimers (Fig. 3), indicating the involvement of (an) unidentified factor(s) driving 100S formation in the absence of both LhpF and RaiA. No significant changes in the ribosome profiles were observed in the *L. pneumophila* Δ*rsfS* and Δ*hflX* mutants (Fig. 3; Fig. S3), analogously to studies using the corresponding *S. aureus* mutant strains (37). However, overexpression of *hflX* led to increased ribosome dissociation (Fig. 3; Fig. S3), reflecting its conserved role in ribosome splitting (36, 37). Future studies will aim at identifying the unknown factor(s) driving 100S formation in the absence of both LhpF and RaiA. To this end, comparative proteomics of purified 100S and 70S ribosomes produced by *L. pneumophila* wild-type or Δ*lhpF*Δ*raiA* double mutant bacteria could be performed.

Ribosome hibernation factors are essential for long-term starvation survival, with the Δ*lhpF*Δ*raiA* double mutant exhibiting the most severe culturability defect (Fig. 4A). Notably, overexpression of *lhpF* impaired regrowth after starvation more than the loss of only *lhpF* or *lhpF* and *raiA* (Fig. 4B), suggesting that gene/protein dosage is essential for the tight regulation of ribosome hibernation and efficient regrowth when conditions improve. The finding that pLhpF but not pRaiA restored regrowth of the double mutant also highlights the importance of maintaining appropriate LhpF protein levels (Fig. 4C). Moreover, this finding might also reflect the vastly different expression profile of *lhpF* and *raiA*, the former being expressed only in stationary growth phase, and the latter exhibiting a striking double peak pattern being expressed in the logarithmic as well as the stationary phase (Fig. 6A). Finally, the partial complementation observed for the ∆*rsfS::rsfS* but not other reintegration strains further suggests that precise transcriptional control is essential for ribosome hibernation (Fig. 4C).

In general, the lack of hibernation factors minimally affects *L. pneumophila* growth under rich conditions but significantly impairs long-term culturability and regrowth upon starvation (Fig. 4; Fig. S2A and B). This has also been observed for *S. aureus* Δ*lhpF* (29, 55), *B. subtilis* Δ*lhpF* (56, 57), *L. monocytogenes* Δ*lhpF* (28), *Pseudomonas aeruginosa* lacking HPF (58, 59), and *Vibrio cholerae* lacking HPF and RaiA (60). *E. coli* triple mutants lacking RMF, HPF, and RaiA show severe regrowth defects after starvation, correlating with increased ribosome fragmentation (61), and *E. coli* lacking *rsfS* exhibit reduced competitiveness during stationary phase survival and nutrient shifts (34). Further studies will address whether and to what extent the culturability of starved *L. pneumophila* ribosome hibernation factor mutants correlates with the viability of the strains (62).

The deletion or overproduction of *L. pneumophila* ribosome hibernation factors affects pathogen-host interactions, including efficient uptake and intracellular replication (Fig. 5; Fig. S5), as well as the production of virulence factors such as the retromer interactor RidL and the ubiquitin ligase SidC (Fig. 6C and D). Similarly, *L. monocytogenes* Δ*lhpF* is impaired for virulence and represents a hyper-hemolytic strain, with the hemolysin listeriolysin O (LLO) being preferentially translated during late exponential growth and stationary growth (28). Moreover, using animal models, ribosome hibernation factors have been implicated in the virulence of *S. aureus* (37) and *L. monocytogenes* (28).

In *L. pneumophila*, perturbing ribosome hibernation increased susceptibility toward not only ribosome targeting antibiotics but also multiple other classes (Fig. 7A). While for tetracycline (targets 30S ribosomal subunit) and ampicillin (cell wall synthesis inhibitor), all hibernation factor mutants showed decreased tolerance, for erythromycin (targets 50S ribosomal subunit), ciprofloxacin (DNA gyrase inhibitor), and rifampicin (RNA polymerase inhibitor), the effects were restricted to the *lhpF* booster and *raiA* overexpression strains. Similar sensitivities to antibiotics (gentamicin and ciprofloxacin) have been previously reported for wild-type *L. pneumophila* (63). Ribosome hibernation factors have also been implicated in antibiotic tolerance of *L. monocytogenes* Δ*lhpF* (43) or *P. aeruginosa* in biofilms, where slow-growing cells showed increased tolerance to ciprofloxacin and the ribosome-targeting aminoglycoside tobramycin, accompanied by elevated transcript levels of *rmf* and *hpf* (21). Similarly, *E. coli* mutants lacking *rmf*, *hpf*, or *raiA* were more susceptible to ciprofloxacin and the β-lactam ampicillin (42). Finally, an overlap between the binding sites of tetracycline at the 30S ribosomal subunit and *S. aureus* LhpF has been reported (32).

Intriguingly, the *L. pneumophila* ribosome hibernation factors are also implicated in several aspects of phenotypic heterogeneity, such as the formation of a population of non-growing intracellular bacteria or of motile, transmissive bacteria (Fig. 7B through D). In previous studies, we have established that the Lqs system, particularly the autoinducer synthase LqsA, is implicated in the regulation of phenotypic heterogeneity in phagocytic host cells (46, 47), as well as in sessile *L. pneumophila* forming microcolonies (64). In these studies, persistence, infection, and motility were found to be traits of *L. pneumophila*, which are subject to phenotypic heterogeneity. Analogously, *P. aeruginosa* Δ*rmf* formed fewer slow-growing, viable cells deeply embedded in biofilms (21), and in *E. coli*, the simultaneous deletion of *rmf*, *hpf*, and *raiA* led to heterogeneous colony sizes after starvation, directly linking hibernation to single-cell regrowth dynamics and phenotypic diversity (61). Given the tight regulation and sub-stoichiometric abundance of ribosome hibernation factors relative to ribosomes, it is likely that the protective effect of hibernation takes place in only a subset of cells, and, accordingly, ribosome hibernation drives phenotypic heterogeneity and contributes to persistence (42, 65).

In conclusion, this study reveals unique features of ribosome hibernation in *L. pneumophila*, including the exclusive formation of 100S ribosomes during the exponential phase. Hibernation factors are essential for long-term starvation survival, regrowth, efficient infection, and the regulation of virulence and phenotypic heterogeneity. Strikingly, both deletion and overexpression of ribosome hibernation factors caused strong phenotypes, emphasizing that not only the presence but also their precise levels critically shape bacterial fitness. Our findings highlight the critical role of ribosome hibernation factors in *L. pneumophila* physiology, persistence, and virulence, offering novel insights into the adaptive strategies of this pathogen under environmental and host-associated stress conditions. Future studies will address the molecular mechanisms involved in protecting the ribosomes from degradation and ribosome “awakening” when growth conditions improve.

## MATERIALS AND METHODS

For details, see the supplemental text.

### Bacteria, bioinformatics, and molecular cloning

*L. pneumophila* strains (Table S1) were grown on CYE agar plates at 37°C for 3–4 days followed by liquid cultures in *N*-(2-acetamido)-2-aminoethanesulfonic acid (ACES)-buffered yeast extract (AYE) medium for 24 h or in minimal defined medium (MDM) (66, 67) for 28 h at 37°C on a wheel (80 rpm).

To identify possible *L. pneumophila* hibernation factors, a BLASTp analysis of known ribosome hibernation factors from *S. aureus* and *E. coli* against the *L. pneumophila* Philadelphia-1 proteome was performed. Predicted protein structures of ribosome hibernation factors from *S. aureus*, *E. coli*, and *L. pneumophila* were generated using the AlphaFold Monomer v2.0 pipeline (68, 69).

The plasmids used and generated in this study are listed in Table S1, and the primers used for PCRs are listed in Table S2. Cloning was performed according to standard protocols. The ribosome hibernation mutants ∆*lhpF (lpg1206*), ∆*raiA (lpg0476),* ∆*rsfS (lpg1377*), and ∆*hflX (lpg0010*) were generated by double homologous recombination as described in (51, 70), replacing the genes with a kanamycin resistance (KanR) cassette. The DKO strain ∆*lhpF*∆*raiA* was generated via double homologous recombination, replacing the gene *raiA* with a gentamycin resistance (*GenR*) gene in the ∆*lhpF::KanR* single mutant. The genomic deletions and lack of other mutations in the mutant strains were verified by whole-genome sequencing.

### Sucrose density gradient analysis and fractionation

For the sucrose density gradient analysis, equal amounts of samples (adjusted to A_260_ = 4.0) were layered onto a 5%–25% (wt/vol) sucrose density gradient containing 50 mM Tris-HCl, pH 7.4, 50 mM NH_4_Cl, 12 mM MgCl_2_, and 1 mM DTT, equilibrated with a Gradient Master 108 (BioComp Instruments). The gradient was centrifuged at 39,000 rpm for 3 h in a swing-out rotor (SW41; Beckman Coulter or TH-641; Thermo Scientific) and analyzed at 254 nm using a density gradient fractionator (Foxy R1 fraction collector, equipped with a UA-6 detector; Teledyne Isco). If needed, fractions of eight drops corresponding to approximately 600 µL were collected for further analysis.

The resulting riboprofiles were scanned, re-traced using CorelDraw, and evaluated using the line graph analysis tool in Fiji. The subsequent x- and y-values were imported as line graphs to Prism, where the corresponding AUC of the different ribosomal sub-populations was quantified and normalized to the total ribosomal population of the respective profile, and the ratio of the 100S to 70S ribosomal populations was calculated.

Ribosomes from sucrose density gradient fractions were visualized by negative stain electron microscopy as described (71).

### Analysis of phagocytosis, intracellular replication, and phenotypic heterogeneity of *L. pneumophila*

Phagocytosis of *L. pneumophila* by *A. castellanii* was analyzed as published (40) by flow cytometry using GFP-producing bacteria. Intracellular replication of *L. pneumophila* in *A. castellanii* and RAW 264.7 macrophages was determined as published (72). Phenotypic heterogeneity was analyzed by flow cytometry using the Timer and the P*_flaA_-gfp* dual fluorescence reporter system in bacteria, as published (46, 47).

### Statistics and usage of artificial intelligence

Statistics were determined by one-way or two-way ANOVAs on the means and standard deviations of at least three replicates. The statistical analysis was performed using the GraphPad Prism software (Version 9.5.1), and differences were deemed statistically significant when the *P*-value was less than 0.05. Artificial intelligence (AI) tools (ChatGPT-4 and -5) were only used to improve the language, accessibility, or quality of human-generated text.

## Data Availability

All data are contained in the article or have been deposited in the European Nucleotide Archive (ENA) at EMBL-EBI, accession number PRJEB104262.

## SUPPLEMENTAL MATERIAL

Supplemental material mbio.03762-25-s0001.pdf Supplemental text, Figures S1-S7, and Tables S1 and S2.

ASM does not own the copyrights to Supplemental Material that may be linked to, or accessed through, an article. The authors have granted ASM a non-exclusive, world-wide license to publish the Supplemental Material files. Please contact the corresponding author directly for reuse.
