# Supplementary material for: Regulation of ribosome hibernation controls Legionella survival, infection, antibiotic tolerance, and phenotypic heterogeneity
Source: mBio. 2026 Jan 30;17(3):e03762-25. doi: 10.1128/mbio.03762-25 (PMC12977618; doi:10.1128/mbio.03762-25)
Supplement: Supplemental material — Supplemental text, Figures S1-S7, and Tables S1 and S2. [file mbio.03762-25-s0001.pdf]

SUPPLEMENTAL INORMATION

Regulation of ribosome hibernation controls *Legionella* survival, infection, antibiotics tolerance, and phenotypic heterogeneity

Camille Schmid[1], Selina Natalie Trinkler[1], Elizabeth Teresa Vittori[1], Michaela Oborská-Oplová[1], Vikram Panse[1] and Hubert Hilbi[1*]

[1]*Institute of Medical Microbiology, University of Zürich, Gloriastrasse 30, 8006 Zürich, Switzerland.*

Running Title: Ribosome hibernation in *L. pneumophila*

Keywords: *Acanthamoeba*, amoeba, antibiotics, intracellular pathogens, *Legionella*, macrophage, pathogen vacuole, persistence, phenotypic heterogeneity, ribosome hibernation, starvation, stress response, virulence.

Abbreviations: cAMP, cyclic adenosine monophosphate; CFU, colony forming units; CTD, C-terminal domain; DKO, double knock-out; GFP, green fluorescent protein; HflX, high frequency of lysogenization X; HPF, hibernation promoting factor; Icm/Dot, intracellular multiplication/defective organelle trafficking; LCV, *Legionella*-containing vacuole; LhpF, long hibernation promoting factor; Lqs, *Legionella* quorum sensing; MIC, minimal inhibitory concentration; MOI, multiplicity of infection; NTD, N-terminal domain; (p)ppGpp, guanosine penta-/tetraphosphate; RaiA, ribosome-associated inhibitor A; RMF, ribosome modulating factor; RsfS, ribosome silencing factor S; T4SS, type IV secretion system.

***Correspondence:** E-mail hilbi@imm.uzh.ch,

Tel.: +41 (0)44 634 2650, Fax: +41 (0)44 634 4906

SUPPLEMENTAL TEXT

MATERIALS AND METHODS

Chemicals, bacteria, and eukaryotic cells

All chemicals were from Sigma-Aldrich or Roth, unless stated otherwise. *L. pneumophila* strains (**Table S1**) were grown on CYE agar plates at 37°C for 3-4 days followed by liquid cultures in *N*-(2-acetamido)-2-aminoethanesulfonic acid (ACES)-buffered yeast extract (AYE) medium for 24 h or in minimal defined medium (MDM) (1, 2) for 28 h at 37°C on a wheel (80 rpm). MDM is an ACES-buffered defined medium containing 10 amino acids, including cysteine and 6 mM serine (as carbon and energy source), and salts, including ferric iron. AYE medium and MDM were supplemented with chloramphenicol (Cam, 5 µg/ml) to maintain plasmids if required.

To determine growth characteristics, *Legionella* strains grown in AYE medium or MDM were diluted to an initial OD_{600} of 0.2 in a black clear bottom 96-well plate (200 µL/well) and incubated at the temperatures indicated in the figure legends while orbitally vigorously shaking. Growth was monitored in triplicates by measuring the absorbance at 600 nm (OD_{600}) using a microtiter plate reader (Cytation 5 Hybrid Multi-Mode Reader, Agilent Technologies). For GFP reporter assays, in addition to the bacterial growth, GFP production was monitored in triplicates by measuring fluorescence (excitation, 485 nm; emission, 528 nm; gain, 50). Values are expressed as relative fluorescence units (RFU) or OD_{600}. For intracellular growth assays using RFU, *L. pneumophila* wild-type and mutants were transformed with plasmid pNT28 (3), yielding GFP-producing strains.

A. castellanii (ATCC 30234, laboratory collection) were cultured in proteose, yeast extract, glucose (PYG) medium at 23°C using Bacto proteose peptone (Life Technologies) and Bacto yeast extract (Life Technologies). Murine RAW 264.7 macrophages (ATCC TIB-71,

laboratory collection) were grown in RPMI 1640 medium (Life Technologies) supplemented with 10% fetal calf serum (FCS, Life Technologies) and 1% L-glutamine (Life Technologies) in a humidified atmosphere at 37°C in 5% CO_2.

Bioinformatic analysis of ribosome hibernation factors in *L. pneumophila*

To identify possible *L. pneumophila* hibernation factors, a BLASTp analysis of known ribosome hibernation factors from *S. aureus* and *E. coli* against the *L. pneumophila* Philadelphia-1 proteome was performed (UniProtKB reference + Swiss-Prot, Taxon ID: 272624), using the following query proteins retrieved from UniProt: LhpF (Q2FIN9), RaiA (P0AD49), RsfS (P0AAT6), HflX (P25519). The analysis resulted in the following proteins of interest: Lpg1206 (Q5ZW81), Lpg0476 (Q5ZY96), Lpg1377 (Q5ZVR3) and Lpg0010 (Q5ZZK0). In addition, homologous proteins to the *E. coli* short hibernation promoting factor (HPF, P0AFX0) and ribosomal modulation factor (RMF, P0AFW2) were searched for, yielding Lpg1206 and Lpg0476 with homology to HPF, but no protein homologous to RMF was identified in *L. pneumophila*. An additional BLASTp analysis of the newly identified ribosome hibernation factors from *L. pneumophila* against the proteomes of other *Legionella* species (namely *L. longbeachae*, *L. micdadei*, and *L. anisa*) was also performed (UniProtKB reference + Swiss-Prot, Taxon ID: 450, 451 and 28082).

Predicted protein structures of ribosome hibernation factors from *S. aureus*, *E. coli*, and *L. pneumophila* were generated using AlphaFold Monomer v2.0 pipeline (4, 5) and visualized in PyMOL Molecular Graphics System, v3.0.2 Schrödinger, LLC (6). Additionally, using FoldMason (7), MSA (multiple sequence alignment) LDDT (local distance difference test) values based on the alignment of the predicted protein structures were calculated. Also, MSA of ribosome hibernation factors from *L. pneumophila*, *S. aureus*, *B. subtilis*, *E. coli*, and *V. cholerae* were generated using ClustalO in Jalview v2.11.4.1 (8), with the additional proteins

being the LhpF homolog from *B. subtilis* (P28368), the RaiA homolog from *V. cholerae* (A0A7Z7YBU1), and the short HPF homolog from *V. cholerae* (A0A0X1L3U0).

Molecular cloning

The plasmids used and generated in this study are listed in **Table S1**, and the primers used for PCRs are listed in **Table S2**. Cloning was performed according to standard protocols. Where indicated, backbones were digested with restriction enzymes from Thermo Fisher Scientific or New England Biolabs (NEB) at 37°C for 20-60 minutes. DNA fragments were amplified using Phusion High Fidelity DNA polymerase (Thermo Scientific) with addition of DMSO, purified with the NucleoSpin Gel and PCR Clean-up Kit (Macherey-Nagel) and assembled via Gibson assembly using the NEBuilder HiFi DNA assembly kit (NEB). Competent *E. coli* TOP10 were transformed with the assembled constructs through heat shock (42°C, 50 sec.). Transformants were selected on LB plates containing Cam, kanamycin (Kan) or gentamycin (Gen). Plasmids were isolated using the NucleoSpin Plasmid Mini kit for plasmid DNA (Macherey-Nagel). Colony PCRs of transformed *L. pneumophila* were performed with Taq polymerase. Genomic DNA was isolated using the GenElute Bacterial Genomic DNA kit (Merck). All constructs were verified by DNA sequencing (Microsynth for sanger and/or ONT sequencing, in-house for whole-genome sequencing).

The GFP reporter plasmids pCS012, pCS016, pCS017, pCS018 and pCS019, harboring a transcriptional P_{lhpF}-, P_{raiA}-, P_{rsfS}- or P_{hflX}-*gfp* (ASV) fusion, were constructed by PCR amplification of the (operon) promoters for *lhpf (lpg1206*, 600 bp), *raiA* (*lpg0476-77*, 200 bp and 600 bp), *rsfS (lpg1377*, 600 bp) and *hflX* (*lpg0009-10*, 600 bp) from *L. pneumophila* JR32 genomic DNA using the primer pairs oCS025/026, oCS041/042, oCS039/040, oCS043/044 or oCS045/046 and cloned into the SacI and XbaI sites of pCM009 (9), thereby replacing P_{flaA}.

The ribosome hibernation mutants Δ*lhpF (lpg1206)*, Δ*raiA (lpg0476),* Δ*rsfS (lpg1377)* and Δ*hflX (lpg0010)* were generated by double homologous recombination as described in (3, 10), replacing the genes by a kanamycin resistance (KanR) cassette. The up- and downstream flanking regions of *lhpF* (600 bp each), *raiA* (900 bp each), *rsfS* (900 bp each) and *hflX* (900 bp each) were amplified from genomic DNA of JR32 *L. pneumophila* using the primer pairs oCS029/030 and oCS033/034, oCS079/048 and oCS051/080, oCS081/058 and oCS061/082, and oCS083/068 and oCS071/084. The *KanR* cassette was amplified using the primer pairs oCS031/032, oCS049/050, oCS059/060, and oCS069/070 and the plasmid pPS001 (10) as template. The amplified fragments for the *lhpF* deletion were cloned into *Bam*HI-digested pUC19 (11) (using pPS001 as template) via a 4-fragment Gibson assembly yielding pCS014. For the *raiA*, *rsfS* and *hflX* deletions, the fragments were first assembled via a linear 3-fragment Gibson assembly followed by a PCR amplification step using the primer pairs oCS079/80, oCS081/82 and oCS083/84. These longer fragments were then cloned into *Bam*HI-digested pUC19 (using pCS014 as template) yielding pCS020 or via a 2-fragment Gibson assembly, with PCR amplified pUC19 backbone (using pCS014 as template) using the primer pairs oCS085/86 and oCS087/88, yielding pCS022 and pCS024. The deletion cassettes consisting of the upstream regions, the *KanR* cassette and the downstream regions of the corresponding genes were then amplified in one piece using the primer pairs oCS035/036, oCS053/054, oCS063/064, oCS073/074. To yield the allelic exchange vectors pCS015, pCS021 pCS023 and pCS025, the amplified sequences were cloned into *Bam*HI-digested suicide vector pLAW344 (12) using pPS002 as template (10), which allows counter-selection with the *sacB* gene.

L. pneumophila wild-type JR32 were transformed with pCS015, pCS021, pCS023 and pCS025 by electroporation and grown on CYE plates supplemented with 20 μg/ml Kan. Individual clones were picked, grown in AYE medium supplemented with 20 μg/ml Kan overnight and spotted on CYE plates supplemented with 20 μg/ml Kan and 20-50 mg/ml

sucrose, or 5 µg/ml Cam. After incubation at 37°C for 3-4 days, clones growing on the CYE/Kan/sucrose plates but not on CYE/Cam plates were picked and purified by dilution streaking. Double-cross-over events and thus correct insertions of the *KanR* cassette in the genome of the deletion mutants were confirmed by colony PCR and sanger sequencing of the deletion region. The genomic deletions and lack of other mutations in the mutant strains were verified by whole-genome sequencing (data deposited in the European Nucleotide Archive (ENA) at EMBL-EBI, accession number PRJEB104262; https://www.ebi.ac.uk/ena/browser/view/PRJEB104262).

The DKO strain Δ*lhpF*Δ*raiA* was generated via double homologous recombination, replacing the gene *raiA* by a gentamycin resistance (*GenR*) gene in the Δ*lhpF::KanR* single mutant. The up- and downstream flanking regions of *raiA* (900 bp each) were amplified from genomic DNA of JR32 *L. pneumophila* using the primer pairs oCS053/121 and oCS054/122. The *GenR* gene was amplified using the primer pair oCS119/120 and the plasmid pLS001 (13) as template. To yield the allelic exchange vector pCS036, the fragments were assembled via a 4-fragment Gibson assembly, with PCR amplified pLAW344 backbone (using pCS015 as template) using the primer pair oTJ208/209.

L. pneumophila JR32 Δ*lhpF* were transformed with pCS036 by electroporation and grown on CYE plates supplemented with 10 µg/ml Gen. Individual clones were picked, grown in AYE medium supplemented with 5 µg/ml Gen overnight and spotted on CYE plates supplemented with 10 µg/ml Gen and 20 mg/ml sucrose, or 5 µg/ml Cam. After incubation at 37°C for 3 days, clones growing on the CYE/Gen/sucrose plates but not on CYE/Cam plates were picked, and the process was repeated using 10 µg/ml Gen in the overnight liquid culture. After the second selection round, clones growing on the CYE/Gen/sucrose plates but not on CYE/Cam plates were picked and purified by dilution streaking. Double-cross-over events and thus correct insertions of the *GenR* gene in the genome of the deletion mutants were confirmed by colony

PCR and ONT sequencing of the deletion region. The genomic deletions and lack of other mutations in the DKO strain were verified by whole-genome sequencing (data deposited in the European Nucleotide Archive (ENA) at EMBL-EBI, accession number PRJEB104262; https://www.ebi.ac.uk/ena/browser/view/PRJEB104262).

To construct the complementation plasmids pLhpF (pCS027) and pRsfS (pCS029) harboring *lhpF* and *rsfS* under the control of their native promotors, the corresponding gene regions including their promotors (600 bp upstream) were amplified from genomic DNA of JR32 *L. pneumophila* using the primer pairs oCS091/092 or oCS097/98, respectively. For the complementation plasmids pRaiA (pCS028) and pHflX (pCS030) harboring *raiA* and *hflX* under the control of their native operon promotors (P_{raiA}: *lpg0476-77* (200 bp)) and P_{hflX}: *lpg0009-10* (600bp)), the corresponding genes and their operonic promotors were amplified from genomic DNA of JR32 *L. pneumophila* using the primer pairs oCS093/094 and oCS095/96, or oCS099/100 and oCS101/102, respectively. The PCR fragments were cloned into the MluI and SalI sites of pNT29 (3), to yield complementation plasmids constitutively expressing *gfp*. These plasmids were used for the complementation of the riboprofiles, where they restored the phenotypes of Δ*lhpF,* Δ*raiA* and Δ*lhpF*Δ*raiA* to the parental strain JR32 but were unable to restore the Δ*hflX* phenotype and even showed an overexpression phenotype for Δ*hflX* pHflX (pCS030).

Additionally, a booster strain for *lhpF* was generated, since the promotor activity of *lhpF* appeared very weak based on the reporter construct P_{lhpF}-*gfp* (pCS012). For this, the native promotor of *lhpF* on the complementation plasmid pLhpF (pCS027) was replaced with the operon promotor of *raiA*, which is approx. 10x stronger and not only active in the stationary phase but also in the exponential phase. To construct the *lhpF* booster plasmid pLhpf* (pCS033), the operon promoter of *raiA* (*lpg0476-77* (200 bp)) was amplified via PCR from genomic DNA of JR32 *L. pneumophila* using the primer pair oCS093/107 and cloned into the

pCS027 backbone amplified via PCR using the primer pair oCS108/109. The *lhpF* booster plasmid pLhpf* was transformed into wild-type JR32 *L. pneumophila* resulting in a *lhpF* booster strain constitutively expressing *gfp*.

The phenotypes of the ribosome hibernation mutants could not be complemented using the plasmids pCS027-30, and specifically Δ*raiA* pRaiA (pCS028) showed an overexpression phenotype, which is why a genomic reintegration approach instead of a plasmid-based system was attempted. To this end, the genes were re-introduced into the genome of the single mutant strains by double homologous recombination, splitting the KanR cassette and re-inserting the native promotor, the genes themselves and a GenR cassette at the original places within the genome. For this, the *KanR* cassette was amplified from the negative strand as two fragments (the downstream 3' fragment, 606 bp, lacking an RBS, rendering it non-translatable; and the upstream truncated 5' fragment, 608 bp, with an artificially inserted stop codon at the end) using the primer pairs oST029/020 and oST040/030 and the plasmid pCS022 as template; the *GenR* cassette using the primer pair oST025/039 and the plasmid pLS001 as template; and the gene *lhpF* with its promotor using the primer pair oST023/027 and the complementation plasmid pCS027 as template. To yield the allelic exchange vector pST003, these fragments were assembled via a 5-fragment Gibson assembly with PCR amplified pLAW344 backbone (using the primer pair oST041/042 and pCS015 as template).

To yield the other allelic exchange vectors pST004-6, the genes and their native promotors were amplified from their corresponding complementation plasmids using the primer pairs oST033/034, oST035/036, and oST037/038; and pCS028, pCS029 and pCS030 as templates, respectively. These fragments were then assembled via a 2-fragment Gibson assembly with PCR amplified pLAW344 backbone (using the primer pair oST031/032 and pST003 as template).

L. pneumophila JR32 Δ*lhpF,* Δ*raiA,* Δ*rsfS* and Δ*hflX* were transformed by electroporation with pST003, pST004, pST005 or pST006, respectfully, and the selection was performed as described above for the DKO, with the following modifications: to directly select for co-integrates, the first selection step after the *Legionella* transformation was performed on CYE/Cam plates. Additionally, after the selection rounds in liquid media containing 5-10 µg/ml Gen, the individual clones were not only checked for loss of growth on CYE/Cam plates but also on CYE/Kan plates and picked from CYE plates supplemented with 15 µg/ml Gen and 20 mg/ml sucrose. Double-cross-over events and thus correct re-insertion of the genes into the genome of the deletion mutants was confirmed by colony PCR and ONT sequencing of the genomic regions.

Crude lysate preparation

L. pneumophila wild-type or ribosome hibernation mutants harboring pNT28 (vector control) or complementation plasmids (pCS027, pCS028, pCS029 and pCS030) or booster plasmids (pCS033) were grown in 100 ml AYE supplemented with Cam (5 µg/ml) at 37°C for 18 h or 48 h on a shaker (80 rpm). For polysome-preserving conditions, 30 µg/ml erythromycin was added to the culture, and it was cooled on ice for 5 min. Cells were harvested by centrifugation, washed once in ice cold lysis buffer (10 mM Tris-HCl, pH 7.4, 100 mM NaCl, 30 mM $MgCl_2$, filter sterilized) supplemented with erythromycin (30 µg/ml), and once more with lysis buffer without erythromycin. The bacterial cells were then disrupted by glass-bead lysis in 1 ml of lysis buffer, using 0.2 g of 0.1 mm glass beads (Scientific Industries, SI-BG01) at max speed (6.5 m/s) for 6 × 1 min (FastPrep-24, MP Biomedical), with breaks lasting at least 1 min on ice in between. After two clarification steps of the lysate (ca. 18'000 × *g*, 10 min at 4°C), aliquots of the supernatant corresponding to an A_{260} of 4 were mixed with 50% glycerol to an end-

concentration of 5% glycerol, snap frozen in liquid nitrogen and stored at -80°C until further analysis.

Sucrose density gradient analysis and fractionation

For the sucrose density gradient analysis, 4 units of A_{260} per sample was layered onto a 5–25% (w/v) sucrose density gradient containing 50 mM Tris-HCl, pH 7.4, 50 mM NH_4Cl, 12 mM $MgCl_2$ and 1 mM DTT, equilibrated with a Gradient Master 108 (BioComp Instruments). The gradient was centrifuged at 39,000 rpm for 3 h in a swing out rotor (SW41; Beckman Coulter or TH-641; Thermo Scientific) and analyzed at 254 nm using a density gradient fractionator (Foxy R1 fraction collector, equipped with a UA-6 detector; Teledyne Isco). If needed, fractions of 8 drops corresponding to approx. 600 µl were collected for further analysis

The resulting riboprofiles were scanned, re-traced using CorelDraw and evaluated using the line graph analysis tool in Fiji. The subsequent x- and y-values were imported as line graphs to Prism, where the corresponding AUC of the different ribosomal sub-populations was quantified, and normalized to the total ribosomal population of the respective profile, and the ratio of the 100S to 70S ribosomal populations was calculated.

Gradient fraction precipitation

For Western blot analysis, the sucrose density gradient fractions (as described above) were precipitated with approx. 10% trichloroacetic acid (60 µl) for 30 min at 4°C, and the pellets were washed twice with acetone after incubation for 10 min at -20°C. The acetone-washed pellets were air-dried for 30 min under the fume hood before resuspending them in 60 µl of 1x SDS sample loading buffer supplemented with 1mM NaOH. On a standard 15-well minigel, 15 µl per fraction was loaded per lane and resolved using 10% TGX SDS-PAGE (Bio-Rad) and analyzed by Western blotting.

Negative stain electron microscopy

Ribosomes from sucrose density gradient fractions (as described above) were visualized by negative stain electron microscopy as described (14). For this, 5 µl of the fractions were directly pipetted onto glow-discharged carbon film coated, 300 mesh, copper grids (Science Services) and blotted away with filter paper after 1 min of incubation. After a brief wash with uranyl acetate (Fluka), the samples were stained with uranyl acetate for 1 min. After drying the grids for at least 10 min, they were imaged using a FEI 27 Tecnai Spirit, a 120 kV electron microscope from the University of Zürich Center for Microscopy (ZMB).

Analysis of starvation survival and regrowth

L. pneumophila strains were grown in AYE supplemented with Cam (5 µg/ml) for 24 h at 37°C to stationary phase. The bacteria were washed once and subsequently diluted to an OD_{600} of ca. 0.1 (~2×10^8 bacteria/ml) in 1 ml ACES-buffered H_2O (pH 6.9±0.1) supplemented with Cam (5 µg/ml) to maintain plasmids. From this, 20 µl were used to start a 6-fold dilution series in 180 µl ACES-buffered H_2O+Cam in a 96-well round-bottom plate, sealed with PetriSEAL (Huberlab) and incubated at 37°C. At each timepoint, evaporation droplets were spun down (1000 × *g*, 10 min, RT), *L. pneumophila* bacteria were resuspended by pipetting 10 times and assessed for their culturability by spotting 10 µl of the dilution series onto CYE+Cam plates. For regrowth assays, 200 µl of the bacteria adjusted to OD_{600} of ca. 0.1 were directly distributed into 96-well round-bottom plates, sealed with PetriSEAL (Huberlab) and incubated at 37°C. At the specific timepoints, evaporation droplets were spun down (1000 × *g*, 10 min, RT), *L. pneumophila* bacteria were resuspended by pipetting 10 times and assessed for their regrowth capacity by inoculating 180 µl AYE+Cam with 20 µl starved bacteria in black clear bottom 96-well plates and measuring the OD_{600} using a microtiter plate reader (Cytation 5 Hybrid

Multi-Mode Reader, Agilent Technologies) for 72 h. The lag time was automatically calculated by the Agilent BioTek Gen 5 v3.16 software.

Analysis of phagocytosis by flow cytometry

Phagocytosis of *L. pneumophila* by *A. castellanii* was analyzed as published (15) by flow cytometry using GFP-producing bacteria. Briefly, the amoebae were seeded in PYG in 6-well plates (1×10^6 cells/well), left to adhere at 23°C for 24 h (cells approximately doubled in this time) and placed in Ac buffer. *L. pneumophila* strains producing GFP were diluted in Ac buffer (16) to the desired density and used to infect the amoebae (MOI 1). The infections were synchronized by centrifugation (880 × *g*, 15 min, RT) and incubated at 30°C. After 1 h of incubation, extracellular bacteria were removed by washing once with Ac buffer. Infected host cells were detached by vigorously pipetting at 2 h, 24 h and 48 h post-infection, fixed with 4% PFA for 30 min at RT in the dark, washed once with Dulbecco's phosphate buffered solution (DPBS; Life Technologie) and directly analyzed by flow cytometry.

The GFP-positive amoebae population was quantified using a Fortessa II flow cytometer and Diva software. The host cell population was identified employing forward (FSC, 250 V) and sideward scatter (SSC, 225 V) gating, with a threshold of 200 each, and examined for the GFP signal (Blue 530_30, 350 V). A total of 10’000 events per sample were recorded. The data were analyzed with the software FlowJo. The GFP-positive host cell population was gated using an uninfected control as reference, and the mean GFP signal of the infected amoeba was calculated.

Intracellular replication of *L. pneumophila* in amoebae and macrophages

Intracellular replication of *L. pneumophila* in *A. castellanii* and RAW 264.7 macrophages was determined as published (17). Briefly, the host cells were seeded in PYG or RPMI 1640,

respectively, in black clear bottom 96-well plates (2×10^4 cells/well), left to adhere at 23°C (amoebae) or 37°C in 5% CO_2 (macrophages) for 24 h (cells approximately doubled in this time) and placed in Ac buffer or RPMI 1640, respectively. *L. pneumophila* strains producing GFP were diluted in Ac buffer (*A. castellanii*) or RPMI 1640 (macrophages) to the desired density and used to infect the host cells (MOI 1). The infection was synchronized by centrifugation (500 × *g*, 10 min, RT) and incubated at 30°C and 37°C (*A. castellanii*) or 37°C in 5% CO_2 (macrophages).

Intracellular bacterial replication was assessed by measuring GFP production using a microtiter plate reader (Cytation 5 Hybrid Multi-Mode Reader, Agilent Technologies). Possible extracellular replication was monitored in wells containing only *L. pneumophila* in Ac buffer or RPMI 1640 without host cells.

Western blot analysis

For assessment of RpsA, SidC, and RidL in eighter sucrose density gradient fractions (as described above) or in crude whole cell lysates (100 ng protein per sample, estimated via A_{280}), samples were separated by SDS-PAGE (10% acrylamide gels) and blotted onto nitrocellulose membranes at 0.3 A, 4°C for 90 min. In general, membranes were blocked with 5% milk in TBSTT (RpsA), 3% milk in TBST (SidC) or 3% BSA in TBS (RidL) for 1 h at RT and incubated first for 1 h at RT and then over-night at 4°C with primary antibodies: polyclonal anti-RpsA antibody (1:3000, AS19 4305, Agrisera), polyclonal anti-SidC antibody (1:1000, affinity purified, (15)) and polyclonal anti-RidL antibody (1:5000, affinity purified, (18)). The secondary antibody (Amersham ECL donkey anti-rabbit IgG HRP-linked whole Ab, Invitrogen) was diluted 1:5000 in the respective blocking solutions (except for RidL: 5% milk in TBS was used) and incubated for 1 h at RT. Protein detection was conducted with Westar Sun ECL Substrate (Cyanagen) and the ImageQuant 800 (Amersham).

Analysis of antibiotic tolerance

L. pneumophila strains were grown in AYE supplemented with Cam (5 µg/ml) for 24 h at 37°C to stationary phase. Bacteria were diluted to an OD_{600} of ca. 0.1 (~2×10^8 bacteria/ml) in 1 ml AYE+Cam additionally supplemented or not with tetracycline (100 µg/mL), erythromycin (100 µg/mL), ciprofloxacin (3.2 µg/mL, Thermo Scientific), ampicillin (100 µg/mL) or rifampicin (1 µg/mL), which corresponds to approx. 100 × MIC in broth (19, 20), and incubated for up to 48 h at 37°C on a wheel (80 rpm). At the specific timepoints, 20 µl were used to start a 6-fold dilution series in 180 µl AYE+Cam in a 96-well round-bottom plate and assessed for their culturability by spotting 10 µl of the dilution series onto CYE+Cam plates.

Analysis of phenotypic heterogeneity by flow cytometry

Phenotypic heterogeneity was analyzed by flow cytometry using the Timer and the P_{flaA}-*gfp* dual fluorescence reporter system in bacteria as published (21, 22). Briefly, the *A. castellanii* amoebae were seeded in PYG in 6-well plates (1×10^6 cells/well) and left to adhere at 23°C for 24 h (cells approximately doubled in this time). *L. pneumophila* strains harboring pNP107 or pSN7, respectively, were diluted in Ac buffer to the desired density and used to infect the amoebae (MOI 1). The infections were synchronized by centrifugation (880 × *g*, 15 min, RT) and incubated at 30°C. After 1 h of incubation, extracellular bacteria were removed by washing once with Ac buffer. The infected amoebae were collected 20 h or 48 h post-infection, respectively, lysed with 0.1% Triton TX-100 (Sigma) in 150 mM NaCl for 30 min at RT in the dark, fixed with 4% PFA for 30 min at RT in the dark, washed once with DPBS and directly analyzed by flow cytometry.

The relevant spectral parameters were recorded and quantified using a Fortessa II flow cytometer and Diva software. The bacterial population was identified employing forward (FSC,

650 V) and sideward scatter (SSC, 300 V) gating, with a threshold of 200 each, and examined for the GFP (Blue 530_30, 700 V) and mCherry signal (YG 610/20, 700 V). To accurately determine the fluorescent particle counts and to be able to compare conditions for the Timer reporter, defined resuspension volume and acquisition times were used. Using a constant flow rate, 3 min per sample were recorded, translating to approx. 2000 events per sample. For the dual fluorescence reporter, 10'000 events per sample were recorded. The data was analyzed with the software FlowJo. The Timer-producing bacterial population was gated using an uninfected control as reference, whereas for the dual fluorescence reporter, also an mCherry only control was used. Spectral properties collected using the Timer reporter were analyzed by calculating the Log10[(Blue 530_30 H)/(YG 610/20 H)] for each detected bacterium and defining a growers/non-growers gate in a histogram, whereas for the dual fluorescence reporter, the GFP positive bacterial population was gated using the mCherry only control as reference.

Statistics

Statistics were determined by Student's *t*-tests and one-way or two-way ANOVAs on the means and standard deviations of at least three replicates. The statistical analysis was performed using the GraphPad Prism software (Version 9.5.1), and differences were deemed statistically significant when the *p*-value was less than 0.05.

Usage of Artificial Intelligence (AI)

AI tools (ChatGPT-4 and 5) were only used to improve the language, accessibility, or quality of human-generated text.

SUPPLEMENTAL FIGURES

Figure S1

A

Blast Hit	Factor	Organism	Identity	Score	E-Value	MSA LDDT
Lpg1206	LhpF	*S. aureus*	28.6%	26.6	0.70	0.555
Lpg0476	RaiA	*E. coli*	38.5%	67.0	6e-17	0.792
Lpg1377	RsfS	*E. coli*	43.2%	89.4	1e-25	0.871
Lpg0010	HflX	*E. coli*	56.3%	442	4e-155	0.889

B

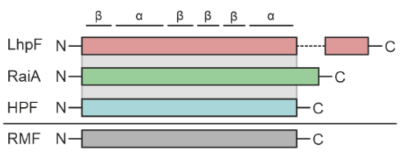

C

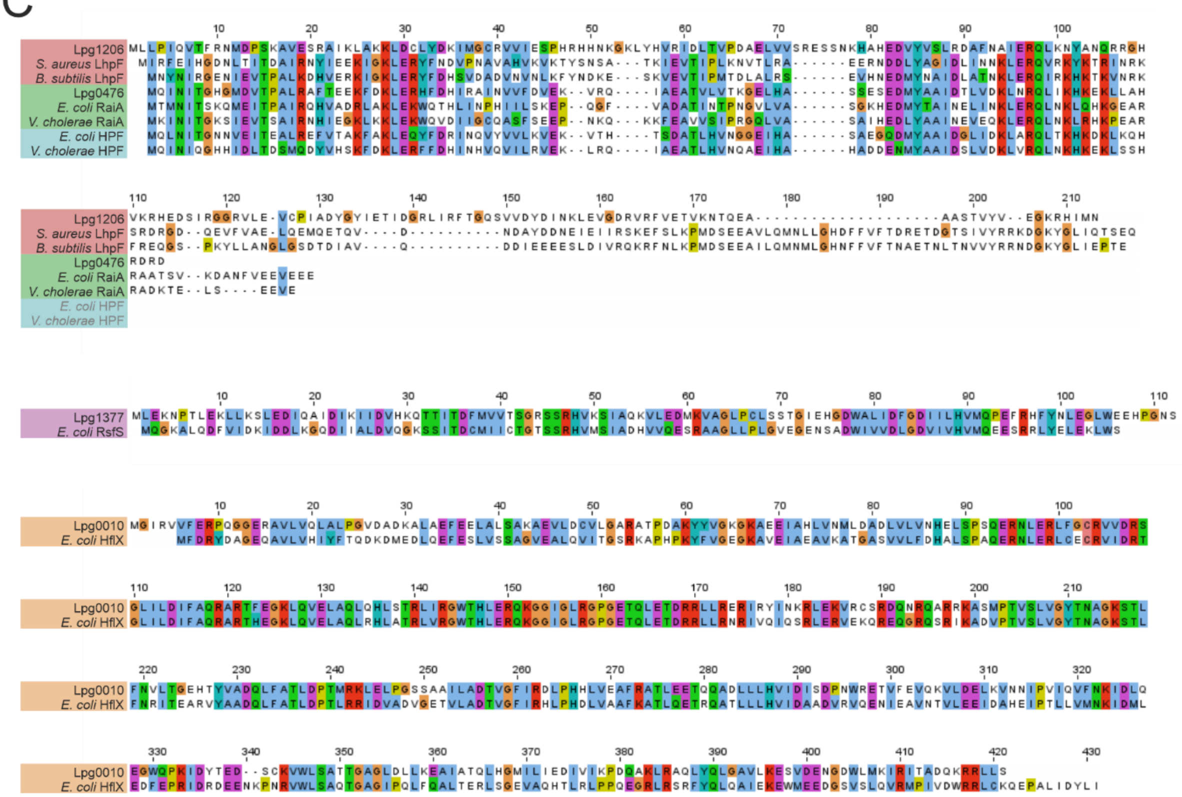

D

Query	Blast Hit	Organism	Identity	Score	E-Value
	Llo1582	*L. longbeachae*	58.9%	209	5e-70
**Lpg1206**	—	*L. micdadei*	—	—	—
	A6J39_011680	*L. anisa*	58.9%	205	1e-68
	Llo2710	*L. longbeachae*	80.4%	160	1e-53
**Lpg0476**	Lmi0823	*L. micdadei*	66.0%	141	4e-46
	A6J39_005895	*L. anisa*	82.5%	160	2e-53
	Llo1467	*L. longbeachae*	65.4%	159	1e-52
**Lpg1377**	Lmi1753	*L. micdadei*	58.6%	145	3e-47
	A6J39_010655	*L. anisa*	65.4%	158	3e-52
	Llo0017	*L. longbeachae*	82.9%	688	0.0
**Lpg0010**	Lmi0009	*L. micdadei*	77.6%	647	0.0
	A6J39_020100	*L. anisa*	84.5%	690	0.0

FIG S1 (overleaf)**. Comparative analysis of ribosome hibernation factors in *L. pneumophila*, *S. aureus*, and *E. coli*.** (A) BLASTp analysis of ribosome hibernation factors from *S. aureus* and *E. coli* against the *L. pneumophila* Philadelphia-1 proteome (UniProtKB reference + Swiss-Prot, Taxon ID: 272624). Query proteins were retrieved from UniProt: LhpF (Q2FIN9), RaiA (P0AD49), RsfS (P0AAT6), HflX (P25519). Results for *L. pneumophila* hits (Lpg1206: Q5ZW81; Lpg0476: Q5ZY96; Lpg1377: Q5ZVR3; Lpg0010: Q5ZZK0) include percent identity, bit score, and e-value. Additionally, MSA (multiple sequence alignment) LDDT (local distance difference test) values are shown, calculated with FoldMason (7) based on the alignment of the predicted structures (Fig. 1A). (B) Structural domain comparison of hibernation factors. LhpF contains a conserved N-terminal domain (NTD, grey) with a β-α-β-β-β-α fold and a dimerization-mediating C-terminal domain (CTD), linked by a variable region. RaiA shares the conserved NTD and includes a partially flexible C-terminal extension, blocking the binding site of RMF, thereby preventing 100S formation. Short HPF consists only of the NTD. RMF is unrelated to HPF and its homologs. (C) Multiple sequence alignment of ribosome hibernation factors from *L. pneumophila*, *S. aureus*, *B. subtilis*, *E. coli*, and *V. cholerae*, generated using ClustalO in Jalview v2.11.4.1 (8). LhpF homologs from *L. pneumophila* (Lpg1206), *S. aureus*, and *B. subtilis* (P28368) are shown in red; RaiA homologs from *L. pneumophila* (Lpg0476), *E. coli*, and *V. cholerae* (A0A7Z7YBU1) in green; and short HPFs from *E. coli* and *V. cholerae* (A0A0X1L3U0) in blue. The alignment of the RsfS homologs from *L. pneumophila* (Lpg1377) and *E. coli* is shown in purple, and that of the HflX homologs from *L. pneumophila* (Lpg0010) and *E. coli* in orange. (D) BLASTp analysis of the ribosome hibernation factors from *L. pneumophila* against the proteomes of other *Legionella* species (UniProtKB reference + Swiss-Prot, Taxon ID: 450, 451 and 28082). Results for hits include percentage identities, bit scores, and e-values.

Figure S2

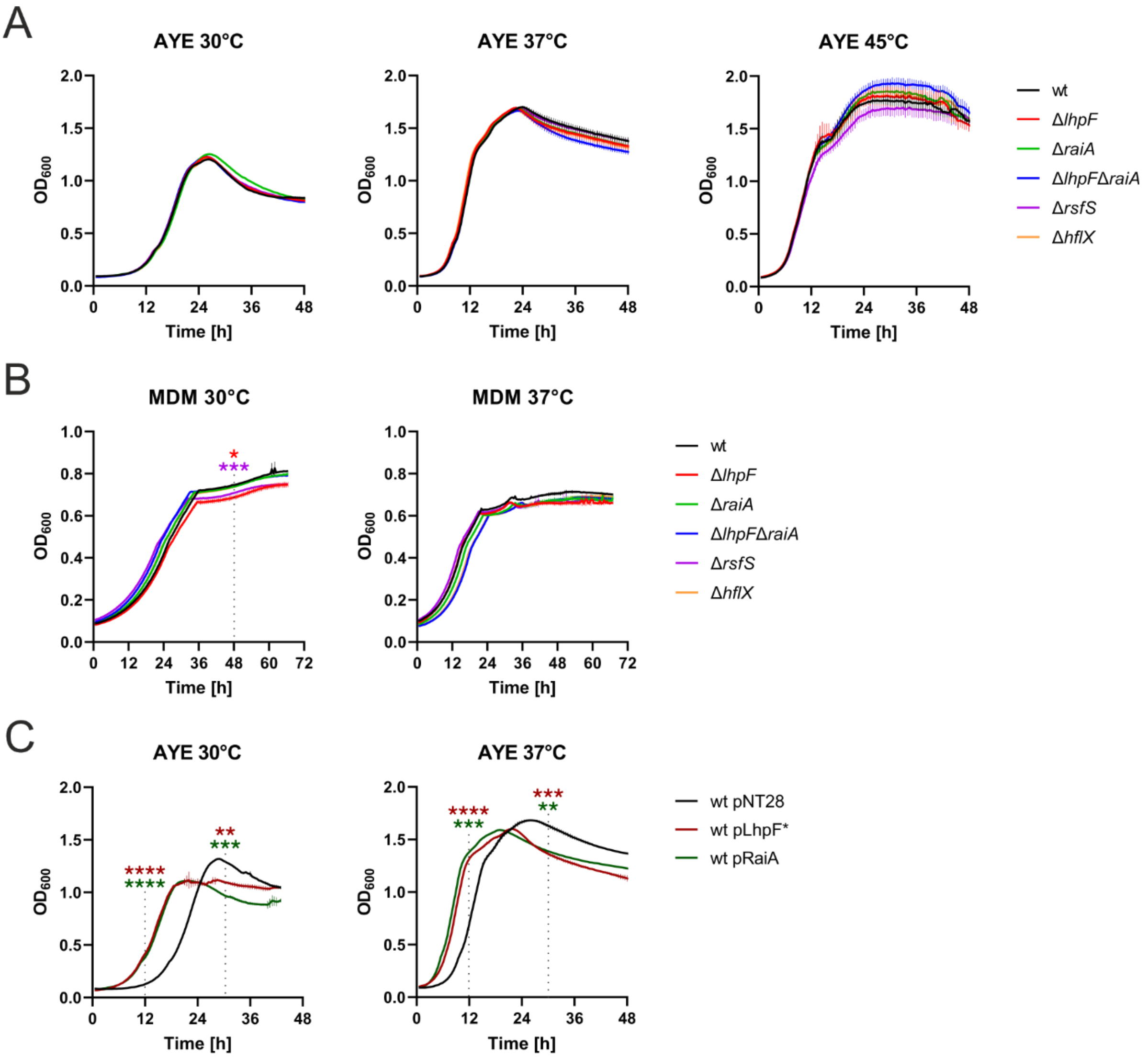

FIG. S2. Growth of *L. pneumophila* ribosome hibernation factor deletion strains in medium. *L. pneumophila* JR32, single and double hibernation factor mutants (Δ*lhpF*, Δ*raiA*, Δ*lhpF*Δ*raiA*, Δ*rsfS*, and Δ*hflX*) were grown in (A) AYE medium or (B) minimal defined medium (MDM) at different temperatures, and OD_{600} was monitored over time. (C) *L. pneumophila* JR32, the *lhpF* booster and the *raiA* overexpression strains, harboring either pNT28, pLhpF* or pRaiA plasmids, were grown in AYE medium at different temperatures, and OD_{600} was monitored over time. Shown are the means and standard deviations of technical triplicates, representative of three biological replicates ($p < 0.05$ *; $p < 0.01$ **; $p < 0.001$ ***; $p < 0.0001$ ****; two-way ANOVA; shown for 48 h, or 12 h and 30 h). Some error bars may not be visible because they are too small and are hidden by the connecting line.

Figure S3

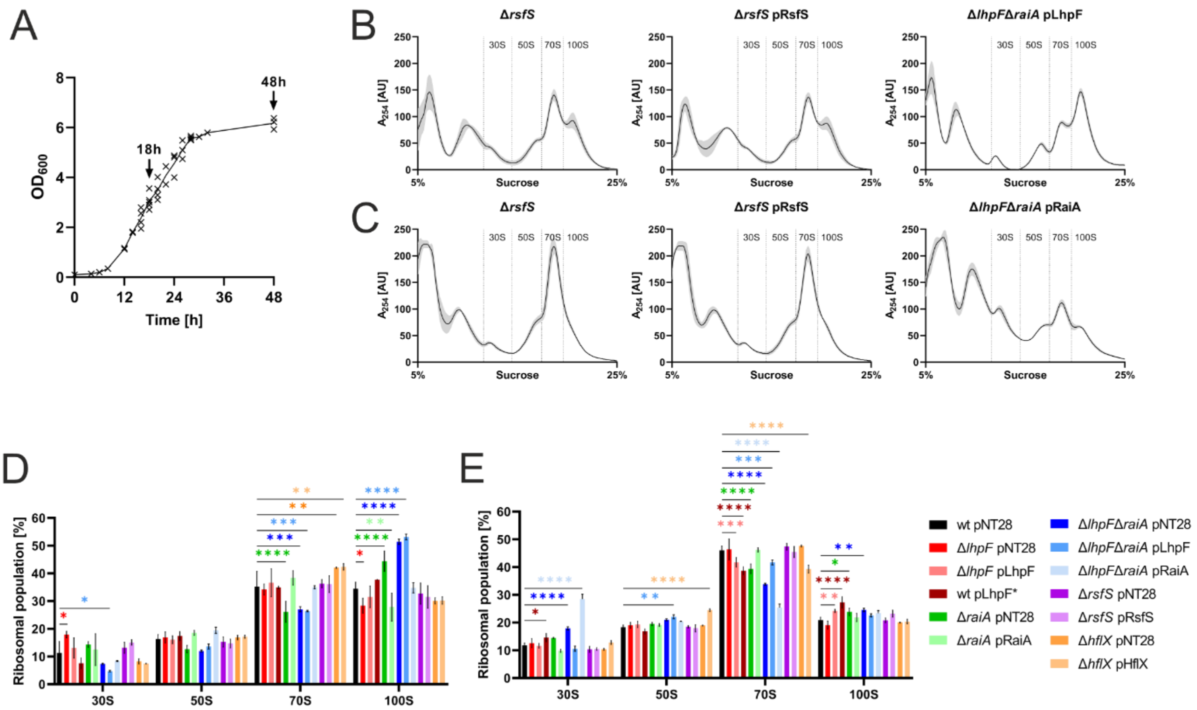

FIG S3. Growth curve of *L. pneumophila* used for crude lysate preparation and riboprofiles of Δ*rsfS*, Δ*lhpF*Δ*raiA* and complemented strains. (A) *L. pneumophila* JR32 harboring pNT28 (vector control) was grown in 100 mL AYE medium (37°C, 80 rpm) and OD_{600} was monitored over time. Shown are single measurements taken from one to five biological replicates and the connecting line of the means. Arrows highlight the 18 h timepoint representing the exponential phase and the 48 h timepoint representing late stationary phase. (B, C) Riboprofiles of *L. pneumophila* Δ*rsfS* harboring pNT28 or pRsfS, and Δ*lhpF*Δ*raiA* harboring eighter pLhpF or pRaiA. Sucrose gradient profiles of ribosomes from *L. pneumophila* whole cell lysates harvested from AYE culture in (B) the exponential phase at 18 h and (C) the late stationary phase at 48 h. The y-axis corresponds to the absorbance at 254 nm (in arbitrary units, AU) of the ribosome population separated on a 5–25% sucrose gradient (x-axis). Graphs show the means and standard deviations (grey shading) of three biological replicates. (D, E) Quantification of the riboprofiles from all strains analyzed, where (D) corresponds to the exponential phase (18 h) and (E) the late stationary phase (48 h) riboprofiles. The area under the curve (AUC) was calculated for each ribosomal sub-population and normalized to the total ribosomal population of the respective profile. Shown are the means and standard deviations of three biological replicates ($p < 0.05$ *; $p < 0.01$ **; $p < 0.001$ ***; $p < 0.0001$ ****; two-way ANOVA).

Figure S4

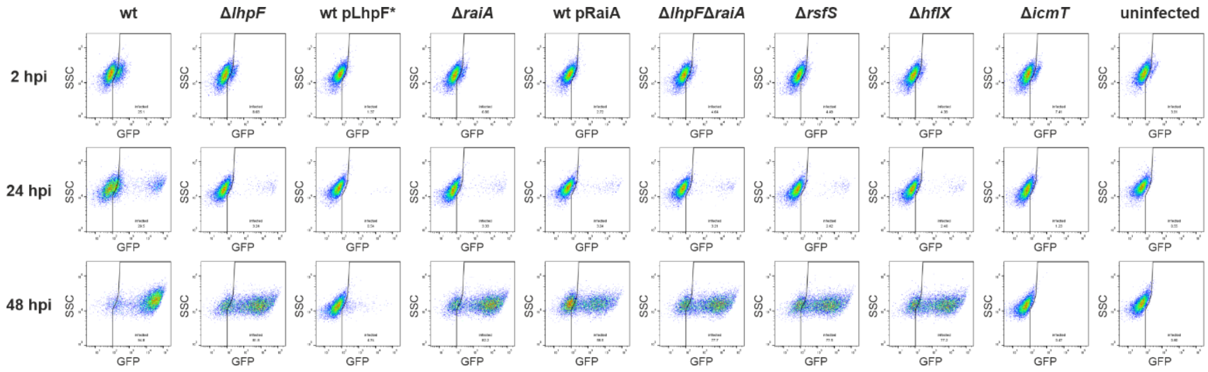

FIG. S4. Flow cytometry gating strategy to assess the number of infected amoebae by *L. pneumophila.* *A. castellanii* amoebae were infected (MOI 1; 2 h, 24 h, 48 h) with GFP-producing *L. pneumophila* JR32, single and double hibernation factor mutants (Δ*lhpF*, Δ*raiA*, Δ*lhpF*Δ*raiA*, Δ*rsfS*, and Δ*hflX*), and the avriulent mutant Δ*icmT* harboring pNT28, or the *lhpF* booster and *raiA* overexpression strains harboring pLhpF* or pRaiA, respectively. Uninfected host cells served as controls and gating aids. Infection rate was assessed by flow cytometry of fixed amoebae. Flow cytometry data are depicted as pseudo color graphs showing side scatter (SSC_A) versus GFP signal intensity of one representative sample of amoebae infected with different *L. pneumophila* strains along with uninfected amoebae at each timepoint.

Figure S5

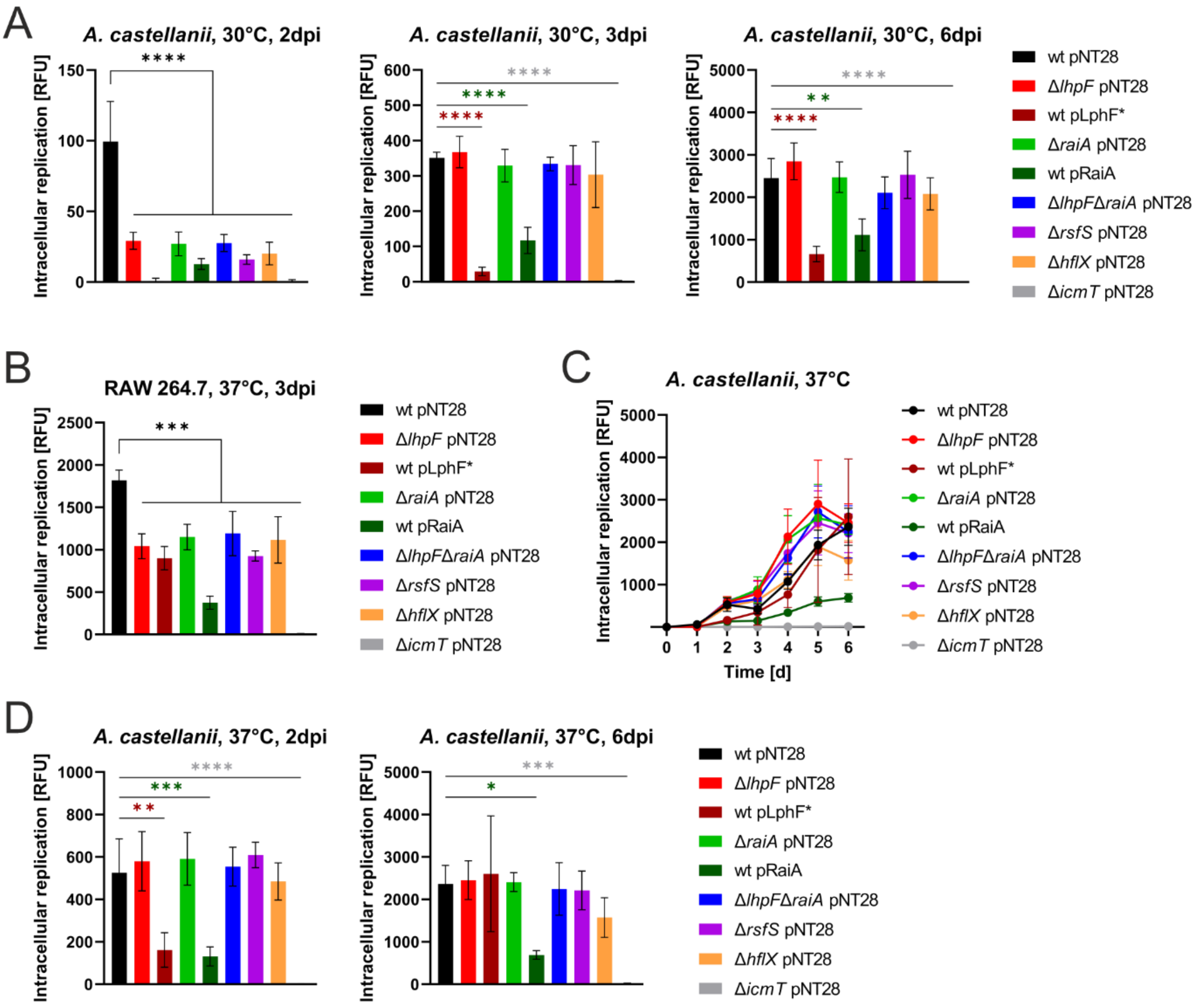

FIG. S5. Zoom in on specific days and statistics for intracellular replication phenotypes and temperature dependence of virulence phenotype of *lhpF* booster strain. GFP-producing *L. pneumophila* JR32, single and double hibernation factor mutants (Δ*lhpF*, Δ*raiA*, Δ*lhpF*Δ*raiA*, Δ*rsfS*, and Δ*hflX*), and the avirulent mutant Δ*icmT* harboring pNT28, or the *lhpF* booster and *raiA* overexpression strains harboring pLhpF* or pRaiA, respectively, were exposed to (A, C, D) *A. castellanii* amoebae at (A) 30°C or (C, D) 37°C or (B) to RAW 264.7 macrophages at 37°C (MOI 1, 6 d). Intracellular replication was assessed by relative fluorescence units (RFU). (A, B, D) Results are depicted as bar graphs at specific timepoints (days post infection (dpi)). Shown are the means and standard deviations of three biological replicates ($p < 0.05$ *; $p < 0.01$ **; $p < 0.001$ ***; $p < 0.0001$ ****; two-way ANOVA). (C) Results are shown over the whole infection period. Shown are the means and standard deviations of three biological replicates.

Figure S6

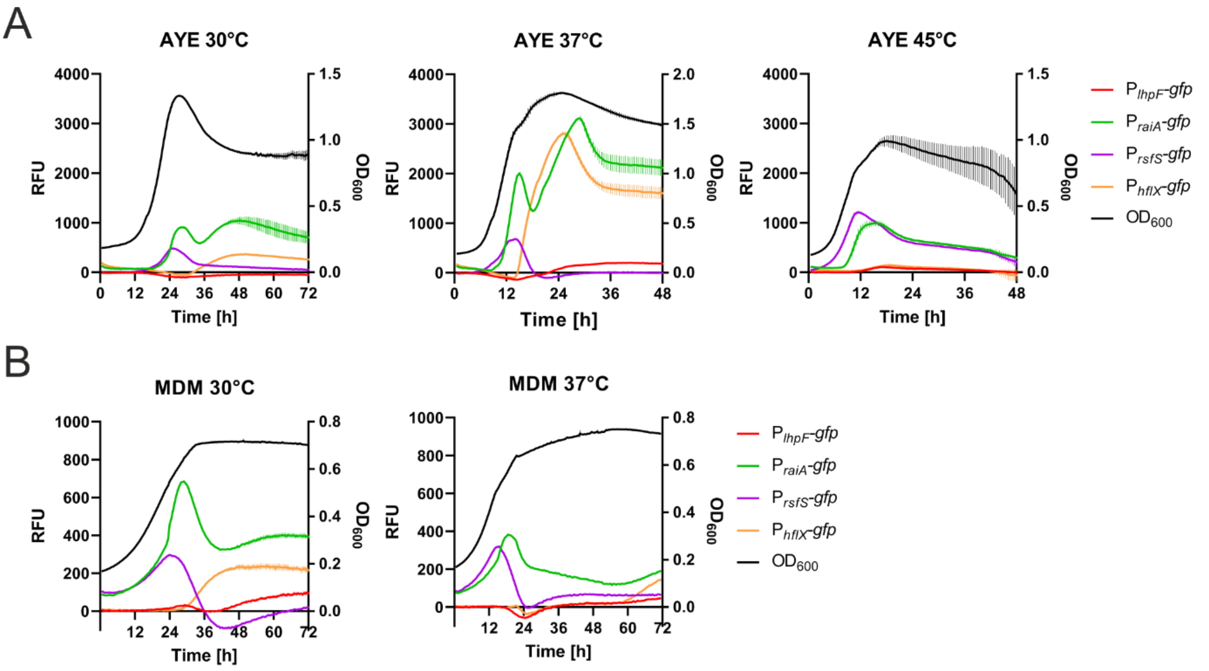

FIG. S6. Expression of *L. pneumophila* ribosome hibernation factors are regulated by temperature and nutrient availability. *L. pneumophila* JR32 harboring P*lhpF*-*gfp* (pCS012), P*raiA*-*gfp* (pCS017), P*rsfS*-*gfp* (pCS018), or P*hflX*-*gfp* (pCS019) were grown in (A) AYE medium or (B) minimal defined medium (MDM) at different temperatures. OD_{600} and GFP fluorescence were measured over time using a microplate reader. Promoter activity is inferred from GFP production levels, denoted as relative fluorescence units (RFU, left y-axis), and one representative OD_{600} curve (right y-axis). Shown are the means and standard deviations of technical triplicates, representative of three biological replicates. Some error bars may not be visible because they are too small and are hidden by the connecting line.

Figure S7

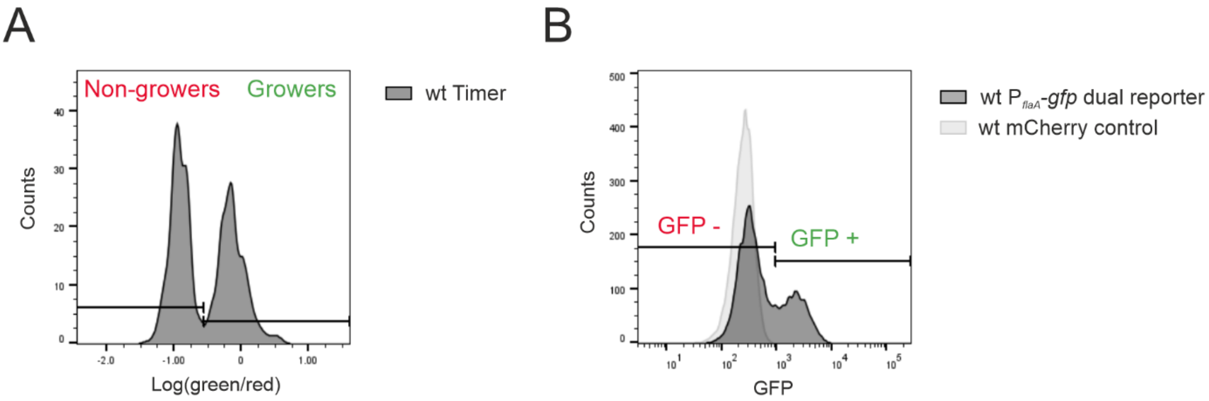

FIG. S7. Flow cytometry gating strategy to assess intracellular phenotypic heterogeneity. (A) *A. castellanii* amoebae were infected (MOI 1, 20 h) with Timer-producing *L. pneumophila* JR32 harboring pNP107. The phenotypic heterogeneity was assessed by flow cytometry in lysates. Uninfected host cell lysate served as control and gating aid. The flow cytometry data are depicted as cell counts versus Log(green/red) signal intensity to discriminate non-growing from growing *L. pneumophila* (one representative sample). (B) *A. castellanii* amoebae were infected (MOI 1, 48 h) with P_{flaA}-*gfp*-expressing *L. pneumophila* JR32 harboring the P_{flaA}-*gfp* dual reporter pSN7, as well as JR32 harboring pNP102 (producing only mCherry as gating control). The phenotypic heterogeneity was assessed by flow cytometry in lysates. Uninfected host cell lysate served as control and gating aid. The flow cytometry data are depicted as cell counts versus GFP signal intensity to identify P_{flaA}-*gfp* expressing bacteria of one representative sample of strain JR32 harboring either the P_{flaA}-*gfp* dual reporter (pSN7) or the mCherry only control (pNP102).

Table S1. Bacterial strains and plasmids used in this study.

Strain or plasmid	Relevant properties [a]	Reference
E. coli		
TOP10		Invitrogen
L. pneumophila		
JR32	*L. pneumophila* Philadelphia-1, serogroup 1, salt-sensitive isolate of AM511	(23)
CS01 (Δ*lhpF*)	JR32 *lpg1206*::KanR	This study
CS02 (Δ*raiA*)	JR32 *lpg0476*::KanR	This study
CS03 (Δ*rsfS*)	JR32 *lpg1377*::KanR	This study
CS04 (Δ*hflX*)	JR32 *lpg0010*::KanR	This study
CS05 (Δ*lhpF*Δ*raiA*)	JR32 *lpg1206*::KanR *lpg0476*::GenR	This study
CS06 (Δ*lhpF::lhpF*)	JR32 *lpg1206*::KanR::$P_{lpg1206}$-*lpg1206*-GenR	This study
CS07 (Δ*raiA::raiA*)	JR32 *lpg0476*::KanR::$P_{lpg0477\text{-}76}$-*lpg0476*-GenR	This study
CS08 (Δ*rsfS::rsfS*)	JR32 *lpg1377*::KanR::$P_{lpg1377}$-*lpg1377*-GenR	This study
CS09 (Δ*hflX::hflX*)	JR32 *lpg0010*::KanR::$P_{lpg0009\text{-}10}$-*lpg0010*-GenR	This study
GS3011 (Δ*icmT*)	JR32 *icmT3011*::KanR	(24)
LM1376 (Δ*rpoS*)	JR32 *rpoS4*::Tn903dGenR	(25)
NT03 (Δ*lqsR*)	JR32 *lqsR*::KanR	(3)
Plasmids		
pCM009	pMMB207C, P_{flaA}-*gfp* (ASV), CamR	(9)
pCS012	pMMB207C, $P_{lpg1206}$-*gfp* (ASV), CamR	This study
pCS014	pUC19, Δ*lpg1206*::KanR	This study
pCS015	pLAW344, Δ*lpg1206*::KanR	This study
pCS016	pMMB207C, $P_{lpg0477\text{-}76,\ 200bp}$-*gfp* (ASV), CamR	This study
pCS017	pMMB207C, $P_{lpg0477\text{-}76,\ 600bp}$-*gfp* (ASV), CamR	This study
pCS018	pMMB207C, $P_{lpg1377}$-*gfp* (ASV), CamR	This study
pCS019	pMMB207C, $P_{lpg0009\text{-}10}$-*gfp* (ASV), CamR	This study
pCS020	pUC19, Δ*lpg0476*::KanR	This study
pCS021	pLAW344, Δ*lpg0476*::KanR	This study
pCS022	pUC19, Δ*lpg1377*::KanR	This study
pCS023	pLAW344, Δ*lpg1377*::KanR	This study
pCS024	pUC19, Δ*lpg0010*::KanR	This study
pCS025	pLAW344, Δ*lpg0010*::KanR	This study
pCS027 (pLhpF)	pMMB207C, Δ*lacI*^{q}, P_{tac}-*gfp* (const.), $P_{lpg1206}$-*lpg1206,* CamR	This study

pCS028 (pRaiA)	pMMB207C, Δ*lacI*^{q}, P_{tac}-*gfp* (const.), $P_{lpg0476}$-*lpg0476,* CamR	This study
pCS029 (pRsfS)	pMMB207C, Δ*lacI*^{q}, P_{tac}-*gfp* (const.), $P_{lpg1377}$-*lpg1377,* CamR	This study
pCS030 (pHflX)	pMMB207C, Δ*lacI*^{q}, P_{tac}-*gfp* (const.), $P_{lpg0010}$-*lpg0010,* CamR	This study
pCS033 (pLhpF*)	pMMB207C, Δ*lacI*^{q}, P_{tac}-*gfp* (const.), $P_{lpg0476}$-*lpg1206,* CamR	This study
pCS036	pLAW344, Δ*lpg0476*::GenR	This study
pLAW344	oriT (RK2), oriR (ColE1), sacB, CamR, AmpR, KanR	(12)
pLS001	pLAW344, Δ*ppgA*::GenR	(13)
pNP102	pMMB207C, Δ*lacI*^{q}, P_{tac}-*mCherry* (const.), CamR	(26)
pNP107	pMMB207C, Δ*lacI*^{q}, P_{tac}-*timer* (const.), CamR	(21)
pNT28	pMMB207C, Δ*lacI*^{q}, P_{tac}-*gfp* (const.), CamR	(3)
pNT29	pMMB207C, Δ*lacI*^{q}, P_{tac}-*gfp* (const.), MCS, CamR	(3)
pPS001	pUC19, *fadL*::KanR	(10)
pPS002	pLAW344, *fadL*::KanR	(10)
pSN7	pMMB207C, Δ*lacI*^{q}, P_{tac}-RBS_{T7}-*mCherry* (const.), P_{flaA}-*gfp* (AAV), CamR	(21)
pST003	pLAW344, $KanR^{down}$-$P_{lpg1206}$-*lpg1206*-GenR-$^{STOP}KanR^{up}$	This study
pST004	pLAW344, $KanR^{down}$-$P_{lpg0477-76}$-*lpg0476*-GenR-$^{STOP}KanR^{up}$	This study
pST005	pLAW344, $KanR^{down}$-$P_{lpg1377}$-*lpg1377*-GenR-$^{STOP}KanR^{up}$	This study
pST006	pLAW344, $KanR^{down}$-$P_{lpg0009-10}$-*lpg0010*-GenR-$^{STOP}KanR^{up}$	This study
pUC19	*oriR* (pBR322), AmpR	(11)

[a] Abbreviations: CamR, chloramphenicol resistance; GenR, gentamycin reistance; KanR, kanamycin resistance; AmpR, ampicillin resistance; const., constitutive; MCS, multiple cloning site; ^{STOP}, stop codon.

Table S2. Oligonucleotides used in this study.

Oligo	Sequence (5'-3') [a]	Comments
oCS025	*CACACAGGAAACAGAATTC*<u>*GAGCTC*</u>TCCAGTCACCTTTGAACTTG	Amplification of P_{lhpF} (600bp), overlap to pCM009 (fo)
oCS026	*GCTCATATGTATATCTCCTTCTTAAA*<u>*T*CTAGAT</u>TTTAACCTCCCTTATGTTTGATTATG	Amplification of P_{lhpF} (600bp), overlap to pCM009 (rev)
oCS029	*GAGCTCGGTACCCGGG*<u>*G*GATCC</u>TCCAGTCACCTTTGAACTTG	Amplification of upstream *lhpF* (600bp), overlap to pUC19 (fo)
oCS030	*GGCAGACCTCAGC*<u>AGATCT</u>TTTTAACCTCCCTTATGTTTGATTATG	Amplification of upstream *lhpF* (600bp), overlap to KanR (rev)
oCS031	*CATAATCAAAACATAAGGGAGGTTAAAA*<u>AGATCT</u>GCTGAGGTCTGCC	Amplification of KanR, overlap to upstream *lhpF* (fo)
oCS032	*GGCCTCTGATACCCCTG*<u>AGATCT</u>GGGAAAGCCACGTTG	Amplification of KanR, overlap to downstream *lhpF* (rev)
oCS033	*CAACGTGGCTTTCCC*<u>AGATCT</u>CAGGGGTATCAGAGGCC	Amplification of downstream *lhpF* (600bp), overlap to KanR (fo)
oCS034	*CAGGTCGACTCTAGAGGATC*<u>*G*GATCC</u>TGTGGGCTTTACTAATTTTTCAAAATC	Amplification of downstream *lhpF* (600bp), overlap to pUC19 (rev)
oCS035	*CCTGCAGCCCGGGG*<u>*G*GATCC</u>TCCAGTCACCTTTG	Amplification of deletion cassette *lhpF*, overlap to pLAW344 (fo)
oCS036	*CGCTCTAGAACTAGTG*<u>*G*GATCC</u>TGTGGGCTTTACTAATT	Amplification of deletion cassette *lhpF*, overlap to pLAW344 (rev)
oCS039	*CAGGAAACAGAATTC*<u>*GAGCTC*</u>GCAAGCGACCCATGG	Amplification of P_{raiA} (600bp), overlap to pCM009 (fo)
oCS040	*GCTCATATGTATATCTCCTTCTTAAA*<u>*T*CTAGA</u>TCAAATCCTCTTTTTTTTTCACTGTC	Amplification of P_{raiA} (600bp), overlap to pCM009 (rev)
oCS041	*CAGGAAACAGAATTC*<u>*GAGCTC*</u>ATTGATAGGCCCCTTTCTTTGG	Amplification of P_{raiA} (200bp), overlap to pCM009 (fo)
oCS042	*GCTCATATGTATATCTCCTTCTTAAA*<u>*T*CTA</u>GATCAAATCCTCTTTTTTTTTCACTGTCTT	Amplification of P_{raiA} (200bp), overlap to pCM009 (rev)
oCS043	*CAGGAAACAGAATTC*<u>*GAGCTC*</u>TTTTCTTGCCAAAAAGCACC	Amplification of P_{rsfS} (600bp), overlap to pCM009 (fo)
oCS044	*GCTCATATGTATATCTCCTTCTTAAA*<u>*T*CTAGA</u>AACTAAGATATATTCCGTCAAAAGGG	Amplification of P_{rsfS} (600bp), overlap to pCM009 (rev)
oCS045	*CAGGAAACAGAATTC*<u>*GAGCTC*</u>TCAGCCAGTCTCAATGC	Amplification of P_{hflX} (600bp), overlap to pCM009 (fo)
oCS046	*GCTCATATGTATATCTCCTTCTTAAA*<u>*T*CTAGA</u>TGCCTACTCCTTGTTGTTATTG	Amplification of P_{hflX} (600bp), overlap to pCM009 (rev)
oCS048	*CGAGGCAGACCTCAGC*<u>AGATCT</u>AAAACCTCCTTTCGTTGACCG	Amplification of upstream *raiA* (900bp), overlap to KanR (rev)

oCS049	*CGGTCAACGAAAGGAGGTTTT*AGATCTGCTGAGGTCTGCC*TCG*	Amplification of KanR, overlap to upstream *raiA* (fo)
oCS050	*GTAAAAGGGTTTGTATTTAAAATAACAT*AGATCTGGGAAAGCCACGTTGTG	Amplification of KanR, overlap to downstream *raiA* (rev)
oCS051	*CACAACGTGGCTTTCCC*AGATCTATGTTATTTTAAATACAAACCCTTTTAC	Amplification of downstream *raiA* (900bp), overlap to KanR (fo)
oCS053	*CCTGCAGCCCGGGG*GATCCAGGATGCGCATCTGTTAATTTAGC	Amplification of deletion cassette *raiA*, overlap to pLAW344 (fo)
oCS054	*GGCCGCTCTAGAACTAGTG*GATCCACAGTTTTAAGGAAGTGGATTATAAGGG	Amplification of deletion cassette *raiA*, overlap to pLAW344 (rev)
oCS058	*CGAGGCAGACCTCAGC*AGATCTAACTAAGATATATTCCGTCAAAAGGG	Amplification of upstream *rsfS* (900bp), overlap to KanR (rev)
oCS059	*CCCTTTTGACGGAATATATCTTAGT*TAGATCTGCTGAGGTCTGCC*TCG*	Amplification of KanR, overlap to upstream *rsfS* (fo)
oCS060	*GATAATGGTAATTTTTAGCATAGATAATG*AGATCTGGGAAAGCCACGTTGTG	Amplification of KanR, overlap to downstream *rsfS* (rev)
oCS061	*CACAACGTGGCTTTCCC*AGATCTCATTATCTATGCTAAAAATTACCATTATC	Amplification of downstream *rsfS* (900bp), overlap to KanR (fo)
oCS063	*CCTGCAGCCCGGGG*GATCCGTCATTTTATATTTTAAGTTCACCTCAGCTG	Amplification of deletion cassette *rsfS*, overlap to pLAW344 (fo)
oCS064	*GGCCGCTCTAGAACTAGTG*GATCCCACTGGTATGTTTTCAGCGAGTAATAC	Amplification of deletion cassette *rsfS*, overlap to pLAW344 (rev)
oCS068	*CGAGGCAGACCTCAGC*AGATCTACAAGAAATTGATTAGTCTGCCACAG	Amplification of upstream *hflX* (900bp), overlap to KanR (rev)
oCS069	*CTGTGGCAGACTAATCAATTTCTTGT*AGATCTGCTGAGGTCTGCC*TCG*	Amplification of KanR, overlap to upstream *hflX* (fo)
oCS070	*CCGCAGCCTGACTTGATTAT*AGATCTGGGAAAGCCACGTTGTG	Amplification of KanR, overlap to downstream *hflX* (rev)
oCS071	*CACAACGTGGCTTTCCC*AGATCTATAATCAAGTCAGGCTGCGG	Amplification of downstream *hflX* (900bp), overlap to KanR (fo)
oCS073	*CCTGCAGCCCGGGG*GATCCACTCGTGAACGATCTTGAGC	Amplification of deletion cassette *hflX*, overlap to pLAW344 (fo)
oCS074	*GGCCGCTCTAGAACTAGTG*GATCCAACTGTATTCGATTGGCGAACAAA	Amplification of deletion cassette *hflX*, overlap to pLAW344 (rev)
oCS079	*CGGCCAGTGAATTCGAGCTCGGTACCCGGGGG*GATCCAGGATGCGCATCTGTTAATTTAGC	Amplification of upstream *raiA* (900bp), overlap to pUC19 (fo)
oCS080	*GCATGCCTGCAGGTCGACTCTAGAGGATCGG*ATCCACAGTTTTAAGGAAGTGGATTATAA	Amplification of downstream *raiA* (900bp), overlap to pUC19 (rev)
oCS081	*AGTGAATTCGAGCTCGGTACCCGGGG*GATCCGTCATTTTATATTTTAAGTTCACCTCAGC	Amplification of upstream *rsfS* (900bp), overlap to pUC19 (fo)

oCS082	*AAGCTTGCATGCCTGCAGGTCGACTCTAGAGGATCG*GATCCCACTGGTATGTTTTCAGCG	Amplification of downstream *rsfS* (900bp), overlap to pUC19 (rev)
oCS083	*ACGACGGCCAGTGAATTCGAGCTCGGTACCCGGGG*GATCCACTCGTGAACGATCTTGAGC	Amplification of upstream *hflX* (900bp), overlap to pUC19 (fo)
oCS084	*TGCATGCCTGCAGGTCGACTCTAGAGGATCGG*ATCCAACTGTATTCGATTGGCGAACAAA	Amplification of downstream *hflX* (900bp), overlap to pUC19 (rev)
oCS085	*GATATTCGCCAGCTGAGGTGAACTTAAAATATAAAATGAC*GGATCCCCCGGGTACCGAGC	Amplification of pUC19 backbone, overlap to upstream *rsfS* (rev)
oCS086	*GGAGTATTACTCGCTGAAAACATACCAGTGGG*ATCCGATCCTCTAGAGTCGACCTG	Amplification of pUC19 backbone, overlap to downstream *rsfS* (fo)
oCS087	*GACTGGCTGAAACAACTCCTGCTCAAGATCGTTCACGAGT*GGATCCCCCGGGTACCGAGC	Amplification of pUC19 backbone, overlap to upstream *hflX* (rev)
oCS088	*CCAGCTCGCTTTTGTTCGCCAATCGAATACAGTT*GGATCCGATCCTCTAGAGTCGACCTG	Amplification of pUC19 backbone, overlap to downstream *hflX* (fo)
oCS091	*CGATCGCCCGGGGATCCTCGAGA*CGCGTCCAGTCACCTTTGAACTTG	Amplification of P_{lhpF} (600bp) and *lhpF*, overlap to pNT29 (fo)
oCS092[b]	*CCGTCGATCGGAGCTCTAGA*GTCGATTAGTTCATAATATGGCGTTTACC	Amplification of P_{lhpF} (600bp) and *lhpF*, overlap to pNT29 (rev)
oCS093[b]	*CGATCGCCCGGGGATCCTCGAGA*CGCGATTGATAGGCCCCTTTCTTTGG	Amplification of P_{raiA} (200bp), overlap to pNT29 (fo)
oCS094	*CCTCCTTTCGTTGACCGATTAA*TCAAATCCTCTTTTTTTTTCACTGTCTT	Amplification of P_{raiA} (200bp), overlap to *raiA* (rev)
oCS095	*AAGACAGTGAAAAAAAAAGAGGATTTGA*TTAATCGGTCAACGAAAGGAGG	Amplification of *raiA*, overlap to P_{raiA} (200bp) (fo)
oCS096[b]	*CCGTCGATCGGAGCTCTAGA*GTCGATTAATCCCTGTCTCTATGGGC	Amplification of *raiA*, overlap to pNT29 (rev)
oCS097	*CGATCGCCCGGGGATCCTCGAGA*CGCGTTTTCTTGCCAAAAAGCACC	Amplification of P_{rsfS} (600bp) and *rsfS*, overlap to pNT29 (fo)
oCS098[b]	*CCGTCGATCGGAGCTCTAGA*GTCGATCAGGAGTTACCAGGGTG	Amplification of P_{rsfS} (600bp) and *rsfS*, overlap to pNT29 (rev)
oCS099	*CGATCGCCCGGGGATCCTCGAGA*CGCGTCAGCCAGTCTCAATGC	Amplification of P_{hflX} (600bp), overlap to pNT29 (fo)
oCS100	*CTAATCCCCAAACAAGAAATTGA*TGCCTACTCCTTGTTGTTATTG	Amplification of P_{hflX} (600bp), overlap to *hflX* (rev)
oCS101	*CAATAACAACAAGGAGTAGGCA*TCAATTTCTTGTTTGGGGATTAG	Amplification of *hflX*, overlap to P_{hflX} (600bp) (fo)
oCS102[b]	*CCGTCGATCGGAGCTCTAGA*GTCGATCATGAAAGCAATCGTCGT	Amplification of *hflX*, overlap to pNT29 (rev)

oCS107	*GTCACCTGAATAGGAAGTAACAT*TCAAATCCT CTTTTTTTTTCACTGTC	Amplification of P_{raiA} (200bp), overlap to *lhpF* (rev)
oCS108	*GACAGTGAAAAAAAAAGAGGATTTGA*ATGTTA CTTCCTATTCAGGTGAC	Amplification of pCS027 backbone, overlap to P_{raiA} (200bp) (fo)
oCS109	*CCAAAGAAAGGGGCCTATCAAT*CGCGTCTCGA GGATCCCC	Amplification of pCS027 backbone, overlap to P_{raiA} (200bp) (rev)
oCS119	*CGGTCAACGAAAGGAGGTTTT*AGATCTATGTT ACGCAGCAGCAACG	Amplification of GenR, overlap to upstream *raiA* (fo)
oCS120	*GATATTAAATTAAAGTAAAAGGGTTTGTATTTAA AATAACAT*AGATCTTTAGGTGGCGGTACTTG GG	Amplification of GenR, overlap to downstream *raiA* (rev)
oCS121	*CGTTGCTGCTGCGTAACAT*AGATCTAAAACC TCCTTTCGTTGACCG	Amplification of upstream *raiA* (900bp), overlap to GenR (rev)
oCS122	*CCCAAGTACCGCCACCTAA*AGATCTATGTTAT TTTAAATACAAACCCTTTTACTTTAATTTAA TATC	Amplification of downstream *raiA* (900bp), overlap to GenR (fo)
oST020	*CAAGTTCAAAGGTGACTGGA*TTTGATGACGA GCGTAATGG	Amplification of KanR^{down}, overlap to $P_{lpg1206}$-*lpg1206* (rev)
oST023	*CCATTACGCTCGTCATCAAA*TCCAGTCACCTT TGAACTTG	Amplification of $P_{lpg1206}$-*lpg1206*, overlap to KanR^{down} (fo)
oST025	*GGTAAACGCCATATTATGAACTAA*GTTTAAAC TCGACCTCGAGGG	Amplification of GenR, overlap to $P_{lpg1206}$-*lpg1206* (fo)
oST027	*CCCTCGAGGTCGA*GTTTAAACTTAGTTCATA ATATGGCGTTTACC	Amplification of $P_{lpg1206}$-*lpg1206*, overlap to GenR (rev)
oST029	*TGCAGCCCGGGGGATCC*GCTGAGGTCTGCCT CGTG	Amplification of KanR^{down}, overlap to pLAW344 (fo)
oST030	*CCGCTCTAGAACTAGTGGATCC*GGGAAAGCC ACGTTGTGTC	Amplification of ^{STOP}KanR^{up}, overlap to pLAW344 (rev)
oST031[c]	GTTTAAACTTTGATGACGAGCGTAATGGC	Amplification of generic pST003 backbone (rev)
oST032[c]	GTTTAAACTCGACCTCGAGGG	Amplification of generic pST003 backbone (fo)
oST033[c]	*GCCATTACGCTCGTCATCAAA*GTTTAAACATT GATAGGCCCCTTTCTTTGG	Amplification of $P_{lpg0476}$-*lpg0476*, overlap to pST003 (fo)
oST034[c]	*CCCTCGAGGTCGA*GTTTAAACTTAATCCCTG TCTCTATGGGC	Amplification of $P_{lpg0476}$-*lpg0476*, overlap to pST003 (rev)
oST035[c]	*GCCATTACGCTCGTCATCAAA*GTTTAAACTTT TCTTGCCAAAAAGCACCGTAG	Amplification of $P_{lpg1377}$-*lpg1377*, overlap to pST003 (fo)
oST036[c]	*CCCTCGAGGTCGA*GTTTAAACTCAGGAGTTA CCAGGGTGTTC	Amplification of $P_{lpg1377}$-*lpg1377*, overlap to pST003 (rev)

oST037[c]	*GCCATTACGCTCGTCATCAAA*GTTTAAACTCA GCCAGTCTCAATGCAATTG	Amplification of $P_{lpg0010}$-*lpg0010*, overlap to pST003 (fo)
oST038[c]	*CCCTCGAGGTCGA*GTTTAAACTCATGAAAGC AATCGTCGTTTTTG	Amplification of $P_{lpg0010}$-*lpg0010*, overlap to pST003 (rev)
oST039	*GTTGATGCGAGTGAT***TAA**AGATCTCGGTATC GATAAGCTTGATATCG	Amplification of GenR, overlap to ^{STOP}KanR^{up} (rev)
oST040	*CGATATCAAGCTTATCGATACCG*AGATCT**TTA** ATCACTCGCATCAAC	Amplification of ^{STOP}KanR^{up}, overlap to GenR (fo)
oST041	*GACACAACGTGGCTTTCCC*GGATCCACTAGT TCTAGAGCGG	Amplification of pLAW344, overlap to ^{STOP}KanR^{up} (fo)
oST042	*CACGAGGCAGACCTCAGC*GGATCCCCCGGGC TG	Amplification of pLAW344, overlap to KanR^{down} (rev)
oTJ208	GGATCCACTAGTTCTAGAGCGG	Amplification of generic pLAW344 backbone (fo)
oTJ209	GGATCCCCCGGGCTGCA	Amplification of generic pLAW344 backbone (rev)

[a] Restriction sites are underlined, regions overlapping with destination vectors or other fragments are in italics, the newly inserted STOP codon is bold. [b] Restriction sites (MluI or SalI) are not included. [c] Newly inserted PmeI restriction sites, so pST004-6 can be used as templates for future cloning by excising and replacing the promotor-gene region through PmeI double restriction.

SUPPLEMENTAL REFERENCES

1. Häuslein I, Manske C, Goebel W, Eisenreich W, Hilbi H. 2016. Pathway analysis using (13) C-glycerol and other carbon tracers reveals a bipartite metabolism of *Legionella pneumophila*. Mol Microbiol 100:229-46.
2. Hochstrasser R, Hilbi H. 2022. The *Legionella* Lqs-LvbR regulatory network controls temperature-dependent growth onset and bacterial cell density. Appl Environ Microbiol 88:e0237021.
3. Tiaden A, Spirig T, Weber SS, Brüggemann H, Bosshard R, Buchrieser C, Hilbi H. 2007. The *Legionella pneumophila* response regulator LqsR promotes host cell interactions as

an element of the virulence regulatory network controlled by RpoS and LetA. Cell Microbiol 9:2903-20.

4. Jumper J, Evans R, Pritzel A, Green T, Figurnov M, Ronneberger O, Tunyasuvunakool K, Bates R, Žídek A, Potapenko A, Bridgland A, Meyer C, Kohl SAA, Ballard AJ, Cowie A, Romera-Paredes B, Nikolov S, Jain R, Adler J, Back T, Petersen S, Reiman D, Clancy E, Zielinski M, Steinegger M, Pacholska M, Berghammer T, Bodenstein S, Silver D, Vinyals O, Senior AW, Kavukcuoglu K, Kohli P, Hassabis D. 2021. Highly accurate protein structure prediction with AlphaFold. Nature 596:583-589.
5. Fleming J, Magana P, Nair S, Tsenkov M, Bertoni D, Pidruchna I, Lima Afonso MQ, Midlik A, Paramval U, Žídek A, Laydon A, Kovalevskiy O, Pan J, Cheng J, Avsec Ž, Bycroft C, Wong LH, Last M, Mirdita M, Steinegger M, Kohli P, Váradi M, Velankar S. 2025. AlphaFold protein structure database and 3D-beacons: new data and capabilities. J Mol Biol 437:168967.
6. Schrödinger, LLC. 2015. The PyMOL molecular graphics system, Version 1.8.
7. Gilchrist CLM, Mirdita M, Steinegger M. 2024. Multiple protein structure alignment at scale with FoldMason. bioRxiv doi:10.1101/2024.08.01.606130:2024.08.01.606130.
8. Waterhouse AM, Procter JB, Martin DMA, Clamp M, Barton GJ. 2009. Jalview Version 2—a multiple sequence alignment editor and analysis workbench. Bioinformatics 25:1189-1191.
9. Schell U, Simon S, Sahr T, Hager D, Albers MF, Kessler A, Fahrnbauer F, Trauner D, Hedberg C, Buchrieser C, Hilbi H. 2016. The α-hydroxyketone LAI-1 regulates motility, Lqs-dependent phosphorylation signalling and gene expression of *Legionella pneumophila*. Mol Microbiol 99:778-93.
10. Hüsler D, Stauffer P, Keller B, Bock D, Steiner T, Ostrzinski A, Vormittag S, Striednig B, Swart AL, Letourneur F, Maass S, Becher D, Eisenreich W, Pilhofer M, Hilbi H. 2023.

The large GTPase Sey1/atlastin mediates lipid droplet- and FadL-dependent intracellular fatty acid metabolism of *Legionella pneumophila*. eLife 12:e85142.

11. Norrander J, Kempe T, Messing J. 1983. Construction of improved M13 vectors using oligodeoxynucleotide-directed mutagenesis. Gene 26:101-6.
12. Wiater LA, Sadosky AB, Shuman HA. 1994. Mutagenesis of *Legionella pneumophila* using Tn*903*dll*lacZ*: identification of a growth-phase-regulated pigmentation gene. Mol Microbiol 11:641-653.
13. Swart AL, Steiner B, Gomez-Valero L, Schütz S, Hannemann M, Janning P, Irminger M, Rothmeier E, Buchrieser C, Itzen A, Panse VG, Hilbi H. 2020. Divergent evolution of *Legionella* RCC1 repeat effectors defines the range of Ran GTPase cycle targets. mBio 11:e00405-20.
14. Mears JA, Hinshaw JE. 2008. Visualization of dynamins. Methods Cell Biol 88:237-56.
15. Weber SS, Ragaz C, Reus K, Nyfeler Y, Hilbi H. 2006. *Legionella pneumophila* exploits PI(4)*P* to anchor secreted effector proteins to the replicative vacuole. PLoS Pathog 2:e46.
16. Moffat JF, Tompkins LS. 1992. A quantitative model of intracellular growth of *Legionella pneumophila* in *Acanthamoeba castellanii*. Infect Immun 60:296-301.
17. Hochstrasser R, Kessler A, Sahr T, Simon S, Schell U, Gomez-Valero L, Buchrieser C, Hilbi H. 2019. The pleiotropic *Legionella* transcription factor LvbR links the Lqs and c-di-GMP regulatory networks to control biofilm architecture and virulence. Environ Microbiol 21:1035-1053.
18. Finsel I, Ragaz C, Hoffmann C, Harrison CF, Weber S, van Rahden VA, Johannes L, Hilbi H. 2013. The *Legionella* effector RidL inhibits retrograde trafficking to promote intracellular replication. Cell Host Microbe 14:38-50.

19. Wilson RE, Hill RLR, Chalker VJ, Mentasti M, Ready D. 2018. Antibiotic susceptibility of *Legionella pneumophila* strains isolated in England and Wales 2007–17. J Antimicrob Chemother 73:2757-2761.
20. Dubois J, St-Pierre C. 1999. In vitro activity of gatifloxacin, compared with ciprofloxacin, clarithromycin, erythromycin, and rifampin, against *Legionella* species. Diagn Microbiol Infect Dis 33:261-265.
21. Personnic N, Striednig B, Lezan E, Manske C, Welin A, Schmidt A, Hilbi H. 2019. Quorum sensing modulates the formation of virulent *Legionella* persisters within infected cells. Nat Commun 10:5216.
22. Striednig B, Lanner U, Niggli S, Katic A, Vormittag S, Brülisauer S, Hochstrasser R, Kaech A, Welin A, Flieger A, Ziegler U, Schmidt A, Hilbi H, Personnic N. 2021. Quorum sensing governs a transmissive *Legionella* subpopulation at the pathogen vacuole periphery. EMBO Rep 22:e52972.
23. Sadosky AB, Wiater LA, Shuman HA. 1993. Identification of *Legionella pneumophila* genes required for growth within and killing of human macrophages. Infect Immun 61:5361-5373.
24. Segal G, Shuman HA. 1998. Intracellular multiplication and human macrophage killing by *Legionella pneumophila* are inhibited by conjugal components of IncQ plasmid RSF1010. Mol Microbiol 30:197-208.
25. Hales LM, Shuman HA. 1999. The *Legionella pneumophila rpoS* gene is required for growth within *Acanthamoeba castellanii*. J Bacteriol 181:4879-4889.
26. Steiner B, Swart AL, Welin A, Weber S, Personnic N, Kaech A, Freyre C, Ziegler U, Klemm RW, Hilbi H. 2017. ER remodeling by the large GTPase atlastin promotes vacuolar growth of *Legionella pneumophila*. EMBO Rep 18:1817-1836.
